# Orthogonal Electrochemical Stability of Bulk and Surface in Lead Halide Perovskite Thin Films and Nanocrystals

**DOI:** 10.1021/jacs.4c06340

**Published:** 2024-08-23

**Authors:** Jence T. Mulder, Julius O. V. Monchen, Yan B. Vogel, Cheng Tai Lin, Filippo Drago, Valentina M. Caselli, Niranjan Saikumar, Tom J. Savenije, Arjan J. Houtepen

**Affiliations:** † Optoelectronic Materials Section, Faculty of Applied Sciences, Delft University of Technology, Van der Maasweg 9, 2629 HZ Delft, The Netherlands; ‡ Chemistry Facility, Istituto Italiano di Tecnologia (IIT), Via Morego 30, 16163 Genova, Italy; § Department of Precision and Microsystems Engineering, Faculty of Mechanical Engineering, Delft University of Technology, Mekelweg 2, 2628 CD Delft, The Netherlands

## Abstract

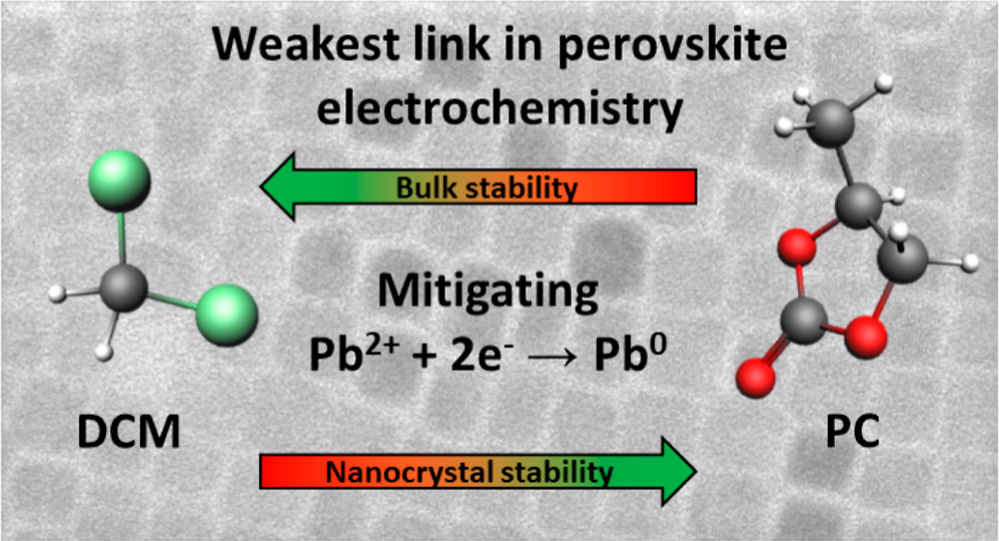

Lead halide perovskites have attracted significant attention for their wide-ranging applications in optoelectronic devices. A ubiquitous element in these applications is that charging of the perovskite is involved, which can trigger electrochemical degradation reactions. Understanding the underlying factors governing these degradation processes is crucial for improving the stability of perovskite-based devices. For bulk semiconductors, the electrochemical decomposition potentials depend on the stabilization of atoms in the lattice–a parameter linked to the material’s solubility. For perovskite nanocrystals (NCs), electrochemical surface reactions are strongly influenced by the binding equilibrium of passivating ligands. Here, we report a spectro-electrochemical study on CsPbBr_3_ NCs and bulk thin films in contact with various electrolytes, aimed at understanding the factors that control cathodic degradation. These measurements reveal that the cathodic decomposition of NCs is primarily determined by the solubility of surface ligands, with diminished cathodic degradation for NCs in high-polarity electrolyte solvents where ligand solubilities are lower. However, the solubility of the surface ligands and bulk lattice of NCs are orthogonal, such that no electrolyte could be identified where both the surface and bulk are stabilized against cathodic decomposition. This poses inherent challenges for electrochemical applications: (i) The electrochemical stability window of CsPbBr_3_ NCs is constrained by the reduction potential of dissolved Pb^2+^ complexes, and (ii) cathodic decomposition occurs well before the conduction band can be populated with electrons. Our findings provide insights to enhance the electrochemical stability of perovskite thin films and NCs, emphasizing the importance of a combined selection of surface passivation and electrolyte.

## Introduction

Lead halide perovskite materials have emerged as a major area of research for optoelectronic applications due to their strong absorbance and intrinsic defect tolerance.^1−9^ Perovskite nanocrystals (NCs) further enhance optical properties and offer tunability through quantum size-effects.^10−15^ Notably, all-inorganic cesium lead halide perovskite NCs (CsPbX_3_, X = Cl^–^, Br^–^, I^–^) exhibit bright photoluminescence (PL) characterized by narrow emission line widths, which is an essential attribute for high color purity lighting and wide color gamut displays.^10,11,16^ In addition, a variety of facile NC synthesis methods exist, offering versatility in surface chemistry and solution-processability.^10,11,17−20^ As such, CsPbX_3_ NCs are promising materials for various optoelectronic applications, including light-emitting devices,^11,21−26^ photocatalysis,^27−35^ and solar cells.^36−38^

A ubiquitous element in all these applications is that charging of the perovskite NCs is involved, either through charge injection, intentional electronic doping, or photoexcitation followed by charge separation.^21,39−42^ Charging can however lead to electrochemical degradation reactions that deteriorate the device’s performance.^43−46^ For example, in the case of CsPbBr_3_ NCs, reduction of Pb^2+^ and oxidation of Br^–^ ions can occur, resulting in cathodic decomposition (eq 1) and anodic decomposition (eq 2), respectively.^45,46^
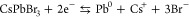
1
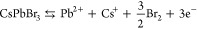
2

The formal potentials of these reactions,  and , depend on the solubility product (*K*_sp_) of the perovskite in the electrolyte solution, as expressed by eqs 3 and 4.^46^
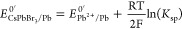
3
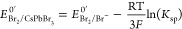
4

Here,  and  represent the formal potentials for the reduction of free Pb^2+^ ions and the oxidation of free Br^–^ ions in the same electrolyte solution, *R* is the gas constant, *T* temperature and *F* the Faraday constant. When CsPbBr_3_ is sparingly soluble in the electrolyte, i.e. *K*_sp_ ≪ 1, cathodic decomposition will take place at more negative potentials than the reduction of free Pb^2+^ ions, whereas anodic decomposition will occur at more positive potentials than the oxidation of free Br^–^ ions.

The positions of the formal potentials of these decomposition reactions relative to the conduction band (CB) and valence band (VB) determine the limits for stable charging of the perovskite by electrons and holes.^43,45,46^ This implies that the stable electrochemical window for charging should widen with decreasing CsPbBr_3_ solubility. In our previous work, we reported quasi-reversible electrochemical charging by holes for CsPbBr_3_ NCs in propylene carbonate (PC), but observed that injection of electrons resulted in cathodic decomposition of the NCs before the CB edge could be reached.^46^ We tentatively attributed the limited cathodic stability of these NCs to the in-gap potential of , whereas  is positioned inside the VB. This implies that CsPbBr_3_ NCs could be reversibly charged by electrons only in electrolytes with a sufficiently low solubility product for CsPbBr_3_, pushing the cathodic decomposition potential into the CB. In continuation to these findings, the present work investigates the relation between the solubility equilibria of CsPbBr_3_ NCs in various electrolytes and their electrochemical stability against cathodic decomposition (eq 1).

Recently a number of scientific publications have addressed the (spectro-)electrochemical characterization of perovskite bulk and NC films.^14,44−55^ These measurements provide information about the optoelectronic properties, such as the location and nature of trap states and the energy levels of the CB and VB.^44−47,49,50,55^ However, unintended electrochemical side reactions can introduce numerous features to the cyclic voltammograms (CVs), thereby adding complexity to the analysis of these measurements.^44−46,55,56^ In relation to this, it is important to note that electrochemistry on perovskites is complicated by their high solubility in nearly all common electrolyte solutions.^45,49,50^

For electrochemical measurements on CsPbBr_3_, this has two important consequences: (i) Dissolved Pb^2+^-complexes in the electrolyte are reduced at electrochemical potentials inside the bandgap, thereby constraining the nonfaradaic electrochemical window, and (ii) cathodic decomposition of CsPbBr_3_ (eq 1) occurs before the CB can be populated with electrons. In the case of NCs, these two effects are further modulated by the presence of surface ligands, such as oleate complexes. The binding equilibrium of these ligands strongly influences the energetics of electrochemical surface reactions and the dissolution of the NCs in the electrolyte. The solubility of Cs^+^ and Pb^2+^ in low polarity solvents will be strongly enhanced by their coordination to oleate ligands commonly employed in perovskite NC synthesis, while Br^–^ ions will be solubilized by oleylammonium ions. It is evident that comprehending the solubility of both the CsPbBr_3_ lattice and the surface ligands is indispensable to obtain a solid understanding of electrochemical processes on CsPbBr_3_ NCs.

In this work, we systematically investigate the cathodic decomposition of CsPbBr_3_ NCs and bulk thin films in various electrolytes through a series of spectro-electrochemical experiments, which combine cyclic voltammetry with measurements of the optical density (OD) and PL. To identify the observed electrochemical features in the CVs, we compare these measurements with the CVs obtained from solutions of Pb^2+^-containing salts, such as PbBr_2_ and Pb-oleate (Pb(OA)_2_). Furthermore, we determined the solubility of bulk CsPbBr_3_ in 26 solvents, along with the solubility of CsOA and Pb(OA)_2_ in the five most pertinent electrolyte solvents. Since the exact nature of the ligand–ion complexes that are involved in the dissolution of CsPbBr_3_ is complex and unknown, we use the solubility of CsOA and Pb(OA)_2_ as a proxy for the relative solubility of surface ligand complexes across different media. For clarity, throughout this text, we refer to general Pb^2+^-surface ligand complexes as Pb-ligand complexes, while using Pb(OA)_2_ to denote the isolatable compound. The solubility analysis contributes to understanding the observed trends in the electrochemical response of CsPbBr_3_ NCs in these electrolytes.

The results show that CsPbBr_3_ is fairly soluble in high-polarity solvents like PC (*K*_sp_ = 10^–12^ mol^5^/L^5^), but only sparingly soluble in lower polarity solvents such as dichloromethane (DCM, *K*_sp_ ≤ 10^–37^ mol^5^/L^5^). The solubility of the surface ligand complexes is, however, orthogonal to CsPbBr_3_, displaying the highest solubility in DCM and only limited solubility in PC. We find that for bulk CsPbBr_3_ films in DCM, the cathodic decomposition potential of −1.7 V vs Fc/Fc^+^ is indeed determined by *K*_sp_ of the ions. In contrast, the electrochemical response of NC films consistently exhibits features around −1.2 V vs Fc/Fc^+^ in all electrolytes; the same is observed for bulk thin films in high-polarity solvents. We attribute these cathodic features at −1.2 V vs Fc/Fc^+^ to the reduction of Pb^2+^-complexes in solution. This is in line with the orthogonal solubility of bulk CsPbBr_3_ and Pb-ligand complexes, which implies that for CsPbBr_3_ NCs a significant amount of dissolved Pb^2+^-complexes is present in all electrolytes.

Ex situ X-ray diffraction (XRD) and X-ray photoelectron spectroscopy (XPS) measurements, combined with in situ spectro-electrochemical measurements are used to verify the assignment of the different electrochemical features. These measurements confirm that both cathodic features, observed at −1.7 and −1.2 V vs Fc/Fc^+^, lead to the formation of metallic Pb^0^. The cathodic feature at −1.7 V is accompanied by a swift dissolution of the CsPbBr_3_ lattice, with the decrease of the perovskite OD proportional to the current, indicative of cathodic decomposition. Conversely, the cathodic features around −1.2 V stem from the reduction of dissolved Pb^2+^-complexes at the electrode. Nonetheless, a noticeable rise in dissolution of the CsPbBr_3_ NCs is associated with this electrochemical feature. This observation suggests a correlation between the reduction of Pb^2+^-complexes in solution and the dissolution of the CsPbBr_3_ NCs, by influencing the dissolution equilibrium.

In addition to this, we observe strong changes to the PL of the CsPbBr_3_ NCs. The PL intensity shows an initial increase as the potential is swept to values negative of the formal potential of the Pb^2+^-complexes in solution, suggesting that significant electrochemical restructuring occurs on the NC surface. Beyond the cathodic decomposition potential of bulk CsPbBr_3_, a rapid and irreversible decline of the PL is observed. These changes in the PL are more pronounced in lower-polarity electrolyte solvents like DCM, where the solubility of Pb-ligand complexes is comparatively higher. Spectro-electrochemical measurements in these electrolytes show a highly irreversible electrochemical response with rapid cathodic decomposition of the NCs. Contrastingly, measurements in higher-polarity electrolytes like PC demonstrate relative stability and reversibility.

Altogether, our spectro-electrochemical experiments indicate that a high Pb(OA)_2_ solubility, which is associated with an increased dissolution of Pb-ligand complexes of the NCs, diminishes their cathodic stability. The current work unravels the electrochemical signatures of CsPbBr_3_ NCs and offers insights to improve their electrochemical stability via a combination of surface ligands and electrolytes. This is particularly relevant for applications that involve a CsPbBr_3_ electrode–electrolyte interface, such as light-emitting electrochemical cells (LECs),^22,23^ photocatalysis,^27−34^ and electrochemical doping.^46^

## Results

**Figure 1:**
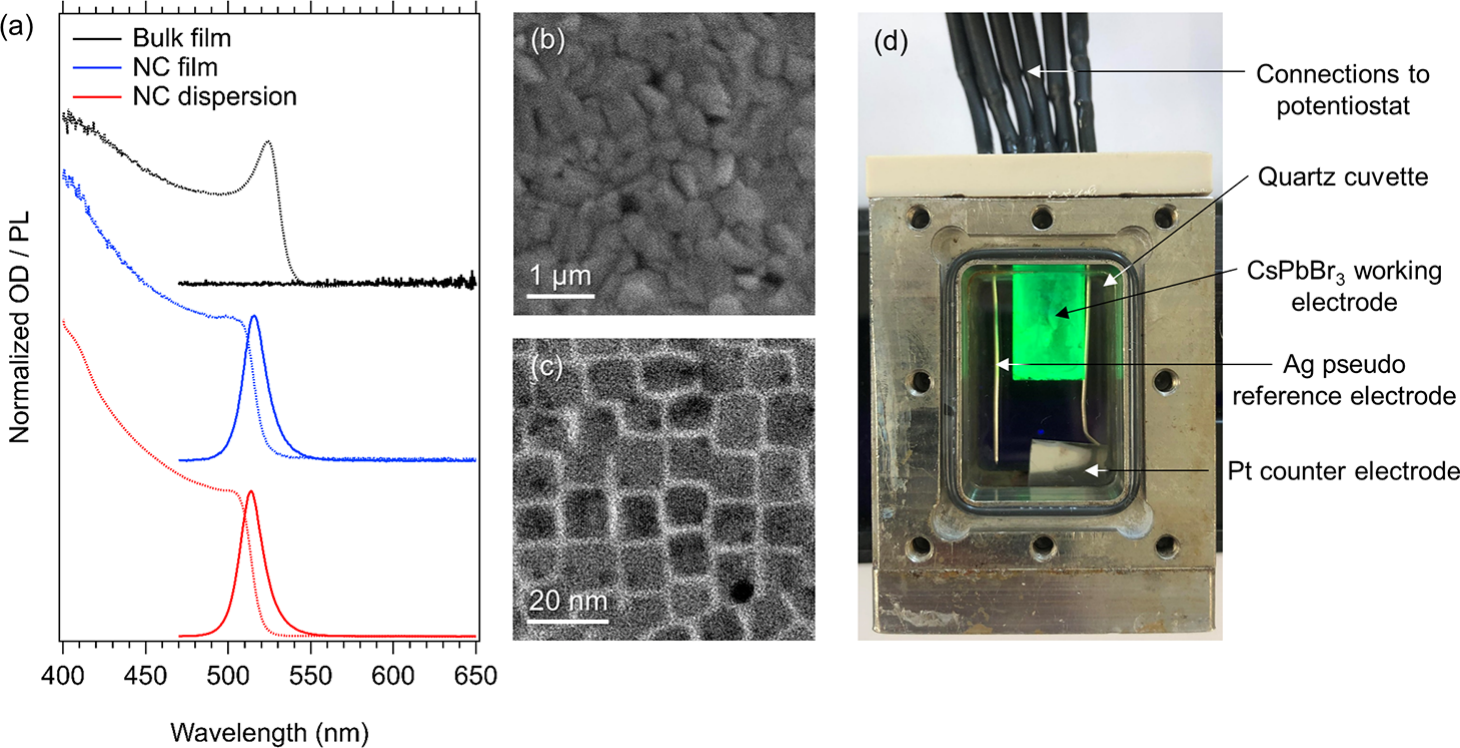
(a) OD (dashed lines) and PL (solid lines) spectra of a CsPbBr_3_ NC dispersion in toluene (red lines), a CsPbBr_3_ NC thin film (blue lines), and a bulk CsPbBr_3_ thin film (black lines) used in this work. (b) A scanning electron microscopy (SEM) image of the thermally evaporated bulk film. (c) Transmission electron microscope (TEM) image of the colloidal NCs. (d) The spectro-electrochemical cell used in this work, featuring a CsPbBr_3_ NC film electrode in a three-electrode arrangement.

In this work we investigate the electrochemical stability of CsPbBr_3_ NCs and bulk crystals, both deposited as thin films on conductive indium tin oxide (ITO) coated glass substrates. Details on the experimental methods are provided in the Supporting Information. Briefly, the NCs were synthesized following a protocol of Imran et al.,^12^ and the NC thin film electrodes were prepared through our previously reported procedure.^46^ The CsPbBr_3_ bulk films were made using a layer-by-layer thermal evaporation method, following Xie et al.^57^ The optical characteristics of the CsPbBr_3_ NC dispersion, NC film and bulk film are shown in Figure 1a, with additional information available in the Supporting Information, Sections SI-7 and SI-8. For electrochemical measurements, an important difference between these two films lies in their microstructure (see electron microscopy images in Figure 1b,c). Simultaneous with the injection of charges, electrolyte ions permeate into the voids of the porous NC film to provide nanoscale charge compensation. In contrast, the charge compensation of the bulk film is limited to the surface of the macroscopic CsPbBr_3_ crystals. The bulk films are consequently expected to function as 2D electrodes, whereas NC films behave as 3D nanoporous electrodes.

### Electrochemical Signatures of CsPbBr_3_ NCs and Bulk Films

**Figure 2:**
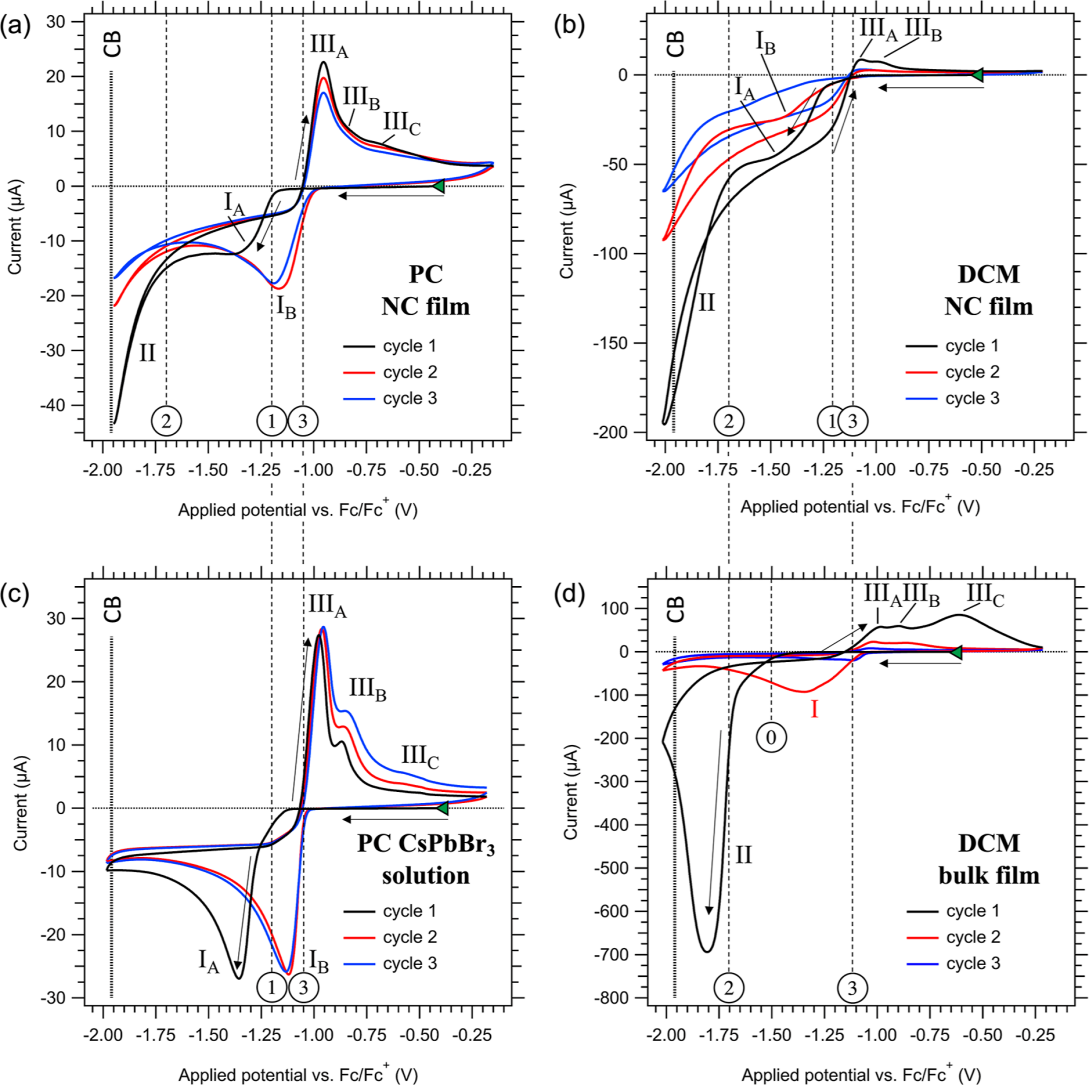
Three CV cycles of a CsPbBr_3_ NC film electrode in (a) PC and (b) DCM, as well as (c) a blank ITO electrode in an undersaturated ionic solution of CsPbBr_3_ in PC, and (d) a bulk CsPbBr_3_ film electrode in DCM. The scan direction is indicated by the black arrows, and Roman numerals denote specific electrochemical features discussed in the text. All CVs begin at the *V*_OC_, indicated by a green triangle, with the potential swept in the negative direction at a scan rate of 10 mV/s. The supporting electrolyte used in all measurements is 0.1 M TBAPF_6_. Circled numbers are referenced for the discussion of the optical effects (vide infra). The sparse dashed lines indicate the corresponding potentials. The dense dashed line indicates the estimated potential of the CB edge.

The electrodes were mounted in a three-electrode electrochemical cell (Figure 1d), and CV measurements were performed in an electrolyte solution of 0.1 M tetrabutylammonium hexafluorophosphate (TBAPF_6_) in either PC or DCM. The resulting CVs are shown in Figure 2. The potential is swept from the open circuit potential (*V*_OC_, indicated by the green triangle) in the negative direction, decreasing to −2.0 V vs Fc/Fc^+^, and then in the positive direction to −0.2 V vs Fc/Fc^+^. All CVs show three cycles to distinguish between reversible and irreversible electrochemical waves. A dashed line indicates the estimated potential of the CB edge, which is established by referencing the VB potential at +0.5 V vs Fc/Fc^+^ as determined in our previous research,^46^ and subtracting the optical bandgap. The position of the CB edge implies that all electrochemical features observed in the CVs occur within the CsPbBr_3_ bandgap.

As displayed in Figure 2, CV measurements of the NC electrodes in PC (Figure 2a) and DCM (Figure 2b) show both similarities and distinct differences. Several key observations can be made.

(1) The CVs for both PC and DCM contain a first cathodic peak (labeled I_A_) starting at approximately −1.2 V vs Fc/Fc^+^ (potential ①) in the first cycle, which shifts to less negative potentials in the subsequent cycles (labeled I_B_).
(2) A second cathodic wave (labeled II) commences at −1.7 V vs Fc/Fc^+^ (potential ②) and shows a steady increase of the current within the scanned potential range in both measurements.
(3) In the reverse scan, multiple anodic waves (labeled III_A,B,C_) are observed, commencing around −1.0 to −1.1 V vs Fc/Fc^+^ (potential ③) in both measurements.
(4) The CVs display a chemically irreversible reduction reaction in both measurements, where the integrated cathodic current (*Q*_cat_) surpasses the integrated anodic current (*Q*_an_) in the reverse scan. Notably, this irreversibility is more pronounced in DCM (*Q*_an_/*Q*_cat_ = 0.03) than in PC (*Q*_an_/*Q*_cat_ = 0.33).

To understand the nature of the observed electrochemical features in these CVs, we conducted identical measurements using a blank ITO working electrode in an undersaturated solution of Cs^+^, Pb^2+^ and Br^–^ ions in PC, obtained by dissolving equimolar amounts of CsBr and PbBr_2_ in the electrolyte. The resulting CV, displayed in Figure 2c, shows striking similarities with the CV of the NC film in PC from Figure 2a. Clearly, both the cathodic peak positions (waves I_A,B_) and the anodic peak positions (waves III_A,B,C_) coincide in the CVs for both measurements. This shows that the origin of these peaks in the CV of the NC film is the reduction of Pb^2+^ ions in solution and the oxidation of metallic Pb^0^ back to Pb^2+^, rather than the cathodic decomposition and reformation of CsPbBr_3_. The electrochemical reduction to metallic Pb^0^ is confirmed by XRD and XPS measurements, as shown in SI-9 and SI-10. In addition, the electrodes visibly exhibit the formation of a dark metallic layer, as is also observed from increased light scattering in the OD measurements (vide infra). Consistent with classical nucleation theory and chronoamperometry measurements described in SI-9, we attribute the potential shift between cathodic waves I_A_ and I_B_ to a nucleation overpotential in the first cycle, which facilitates the formation of Pb^0^-nuclei on the electrode.^58−60^ Since not all Pb^0^ is oxidized in the anodic wave (*Q*_an_/*Q*_cat_ = 0.33), some of the Pb^0^ domains persist throughout the subsequent cycles, such that this nucleation overpotential is observed only in the first cycle. The primary difference between the CVs of the NC films and the blank ITO electrode in a solution of Cs^+^, Pb^2+^ and Br^–^ ions in PC, is the absence of the cathodic wave II in the latter measurement. Hence, we tentatively attribute cathodic wave II to cathodic decomposition of the NCs involving reaction 1 (vide infra).

Cathodic wave II commences at approximately −1.7 V vs Fc/Fc^+^ (potential ②) for both measurements of the NC films (Figure 2a,b). However, the current associated with this wave is roughly four times larger in DCM than in PC. As discussed in more detail below, low-polarity solvents (e.g., DCM) are expected to exhibit much lower CsPbBr_3_ solubilities than high-polarity solvents (e.g., PC), which consequently should lead to a more negative cathodic decomposition potential in the former case. Given this, it is surprising at first glance that the NC film displays a much larger cathodic decomposition current in DCM than in PC. Additionally, wave I, associated with the reduction of Pb^2+^ ions in solution, shows a 2.5-fold larger current in DCM than in PC. We find that both observations can be explained by the orthogonal solubility of CsPbBr_3_ and the Pb-ligand complexes (vide infra), which implies that a significant amount of dissolved Pb^2+^-complexes is present in all electrolytes. We tentatively attribute cathodic wave I in the measurement of the NC film in DCM to the reduction of dissolved Pb-ligand complexes, as CV measurements of a solution of Pb(OA)_2_ in DCM show a cathodic peak at −1.4 V vs Fc/Fc^+^ (see SI-11), similar to the position of wave I in the CV of the NC film.

To investigate the electrochemical response of CsPbBr_3_ in the absence of surface ligands, we performed identical CV measurements on thermally evaporated bulk CsPbBr_3_ films in DCM. The resulting CV is displayed in Figure 2d. Like the CV measurement of the NC film in DCM, the bulk film displays a large cathodic current at approximately −1.7 V vs Fc/Fc^+^, i.e. wave II. Since this wave is consistently present in all CVs involving CsPbBr_3_ electrodes, but not in the CV of a solution of Cs^+^, Pb^2+^ and Br^–^ ions, this reinforces our assignment of wave II as the cathodic decomposition reaction. The cathodic current of wave II in the CV of the bulk film in DCM is ∼3.5 times larger than for the NC film in DCM, and ∼17 times larger compared to that of NC film in PC. This suggests that the cathodic reactivity of bare CsPbBr_3_ surfaces is substantially larger than for passivated NCs, as demonstrated by the rapidly diminishing current in the second cycle and its near disappearance in the third cycle of the CV for the bulk film, indicating that the CsPbBr_3_ has fully decomposed. A key difference between the CVs of the bulk film and NC film is the absence of wave I in the first cycle of the bulk film. This is again a strong indication that wave I is associated with the reduction of Pb^2+^-complexes in solution, which are absent (or present at much lower concentration) for the bulk film in DCM.

### Solubility of CsPbBr_3_ and Ligand Complexes in Various Electrolyte Solvents

To quantify the solubility of CsPbBr_3_ and oleate surface ligand complexes in different electrolyte solvents, we conducted inductively coupled plasma optical emission spectroscopy (ICP-OES) measurements using saturated solutions of CsPbBr_3_, Pb(OA)_2_ and CsOA in PC, DCM, and a large number of other common electrolyte solvents. A full overview of the CsPbBr_3_ solubility in 26 different solvents, along with the solubility of Pb(OA)_2_ and CsOA in a selection of five solvents, can be found in SI-12 to SI-16. In this discussion, we will focus on the solubility of CsPbBr_3_ and Pb(OA)_2_ in five representative electrolyte solvents: PC, DCM, acetonitrile (MeCN), benzonitrile (PhCN) and tetrahydrofuran (THF).

**Figure 3:**
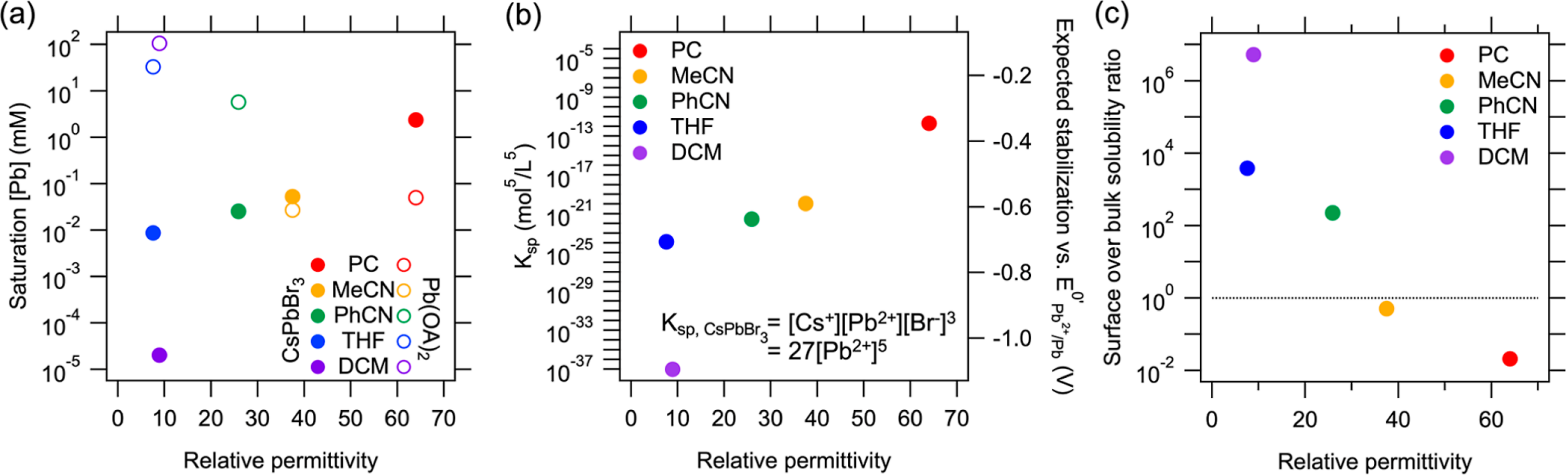
(a) [Pb^2+^] in solutions of CsPbBr_3_ and Pb(OA)_2_ in PC, acetonitrile (MeCN), benzonitrile (PhCN), THF and DCM at saturation, as determined by ICP-OES elemental analysis. (b) The solubility product *K*_sp_ of CsPbBr_3_, estimated from the measured [Pb^2+^] displayed in (a). The right axis shows the expected electrochemical stabilization based on *K*_sp_, as calculated through eq 3. (c) The ratio of the determined molar Pb(OA)_2_ solubility and the molar CsPbBr_3_ solubility. This ratio indicates whether CsPbBr_3_ NCs are stabilized against dissolution primarily by their surface ligands (<1), or by the bulk CsPbBr_3_ solubility (>1). For all subfigures, the data points are plotted versus the relative permittivity of the solvents used.

Figure 3a displays the Pb^2+^ concentration of the CsPbBr_3_ (solid circles) and Pb(OA)_2_ (open circles) solutions at saturation, as determined by ICP-OES measurements (see SI-6 for the measurement details). Assuming a full ionic dissociation of CsPbBr_3_ in these solutions, we approximate its solubility product  Following eq 3, the expected electrochemical stabilization potential of CsPbBr_3_, , is displayed in Figure 3b. As observed, the CsPbBr_3_ solubility in DCM (≤2.03 × 10^–5^ mM) is at least 5 orders of magnitude lower than in PC (∼2.38 mM). The solubility in DCM is so low that no Pb-signal could be recorded above the background noise (<50 ppb) in ICP-OES. The determined molar solubility and *K*_sp_ of ≤10^–37^ mol^5^/L^5^ therefore represent upper limits. In contrast, the measured *K*_sp_ in PC is ∼10^–12^ mol^5^/L^5^. This implies a ≥0.75 V difference for the cathodic decomposition potential of CsPbBr_3_ in PC and DCM. Generally, the solubility of CsPbBr_3_ tends to increase with the relative permittivity of the solvents, as is expected for ionic solutes. The divergence of the CsPbBr_3_ solubility in THF and DCM, despite their similar relative permittivity, might be attributed to the mild Lewis basicity of THF, allowing it to coordinate with Pb^2+^ and Cs^+^ ions in solution.

The opposite trend is observed for the solubility of Pb(OA)_2_ in the same solvents (indicated by the open circles in Figure 3a), serving as a proxy for the relative solubility of Pb-ligand complexes in these media. Pb(OA)_2_ is highly soluble in DCM (∼33 mM) and only sparingly soluble in PC (∼50 μM). This implies that the solubility of CsPbBr_3_ and the Pb-ligand complexes is orthogonal. Figure 3c summarizes these trends by displaying the ratio of the molar solubilities of Pb(OA)_2_ and CsPbBr_3_ in the examined solvents.

This highlights an important complication for electrochemical measurements on ligand-passivated CsPbBr_3_ NCs: In low-polarity solvents (*e.g.*, DCM), the NCs are prone to dissolution of Pb and Cs ligand complexes. Conversely, dissolution of the CsPbBr_3_ lattice occurs in high-polarity solvents (*e.g.*, PC). While the presence of poorly soluble surface ligands hampers the dissolution of the CsPbBr_3_ NC core, we consider that these surface ligands are dynamically bound, allowing the underlying CsPbBr_3_ lattice to dissolve gradually. In other words, Pb^2+^-complexes are inherently introduced in all electrolyte solvents during measurements on NC films. This analysis explains the appearance of wave I in the CVs of the NC films, which we tentatively attributed to the reduction of dissolved Pb^2+^-complexes, and its absence in the case of a bulk film in DCM.

Following these solubility trends, low-polarity electrolyte solvents like DCM have lower CsPbBr_3_ solubilities and, consequently, more negative cathodic decomposition potentials than high-polarity solvents like PC. However, as discussed earlier, the CVs show an increased cathodic reactivity for the NC film in DCM compared to PC. We propose that the cathodic stability of NC films can be severely influenced by dissimilar ligand surface coverages, as a consequence of the varying solubility of oleate ligand complexes in these media. To investigate this further, we recorded the OD and PL spectra of the films in situ during electrochemical cycling. We focus our discussion on the results in PC and DCM, i.e. the two extremes in terms of the solubility of the bulk and surface. Similar experiments were performed in MeCN, PhCN and THF, as reported in SI-22 to SI-24.

### In Situ Spectro-electrochemical Measurement of the OD

**Figure 4:**
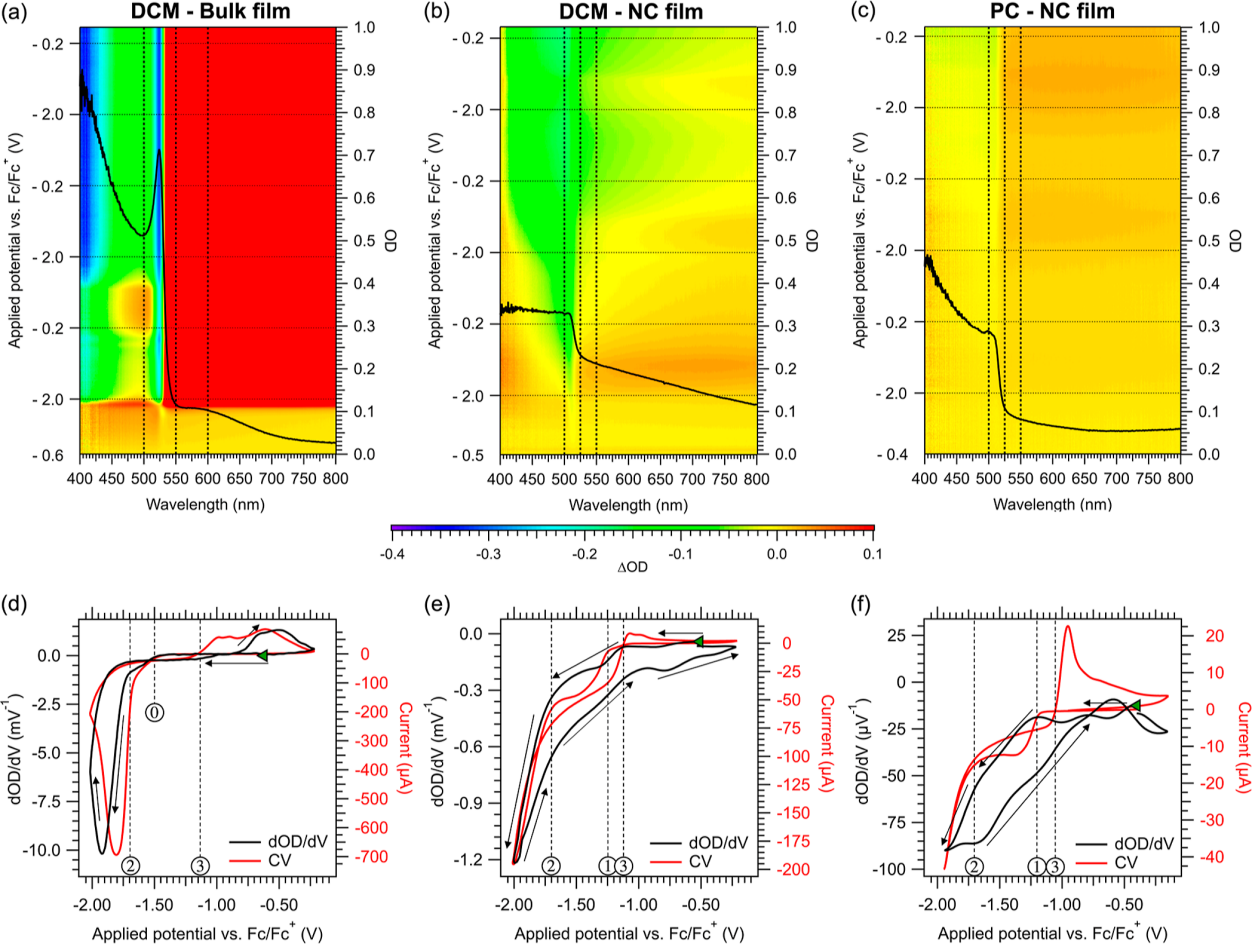
False-color images showing the changes to the OD of (a) a bulk CsPbBr_3_ film in DCM, (b) an NC film in DCM and (c) an NC film in PC, measured in situ during the three CV cycles displayed in Figure 2. Each figure includes an overlay of the initial OD spectrum, along with the wavelength regions (dotted lines) used for averaging the band-edge and sub-bandgap changes. Horizontal dotted lines indicate the turning points of the CV scans. (d) Shows the dOD/dV spectrum (black) and the corresponding first CV cycle (red) for the bulk CsPbBr_3_ film in DCM and (e) the NC film in DCM, and (f) the NC film in PC. The dOD/d*V* spectra are specific for changes to the perovskite absorbance, by correcting the OD at the band-edge for shifts in the baseline at sub-bandgap wavelength. The starting point of the CV at *V*_OC_ (green triangle), the scan direction (black arrows), and the potentials discussed in the text (circled numbers) are indicated in each figure.

Figure 4a–c show false-color images of the change to the OD of the NC- and bulk-films as a function of the applied potential, measured during the CVs displayed in Figure 2. Absorption spectra at selected potentials (horizontal slices of the 2D images shown in Figure 4a–c) are shown in Section SI-27. The bulk CsPbBr_3_ film (Figure 4a) exhibits swift changes in the OD at potentials negative of −1.7 V vs Fc/Fc^+^, coinciding with the onset of cathodic wave II at potential ②. Below the band edge (>550 nm), the OD strongly increases due to scattering by the metallic Pb^0^ that is deposited on the electrode. Simultaneously, a rapid decrease of the OD above the band edge is observed as the CsPbBr_3_ undergoes near-complete cathodic dissolution. The same effects are observed for the NC film in DCM (Figure 4b). The absolute changes in the OD are smaller as the film is thinner, yet the CsPbBr_3_ NC absorption is entirely gone after three CV cycles, similar to the behavior of the bulk film. This is in sharp contrast to the small and relatively reversible changes of the OD for the NC film in PC (Figure 4c). The band edge absorption has decreased by only 20% after three CV cycles, indicating that the cathodic decomposition of the NCs occurs more sluggish. These differences are further highlighted in SI-17 and SI-27, showing the change in OD spectra during and after the CVs.

The onset of the cathodic decomposition reaction should coincide with a decrease in the band-edge absorbance of the CsPbBr_3_ film. No significant changes in the band-edge absorbance are expected at more positive potentials, in the absence of film dissolution. Nonetheless, the analysis of the OD spectra is complicated by shifts in the baseline, as the electrodeposition of metallic Pb^0^ on the electrode results in increased light scattering. To this end, we correct the measured changes in the perovskite band-edge absorbance by subtracting the sub-bandgap changes from the OD, as indicated in Figure 4a–c. For example, for the bulk film in DCM, shown in Figure 4a, we subtract the ΔOD signal averaged between 550 and 600 nm from the ΔOD signal averaged between 500 and 550 nm (dashed vertical lines in Figure 4a). This correction yields a ΔOD signal that is specific for changes in the perovskite absorbance only, as shown in SI-18. Here, we work under the assumption that the baseline offset at sub-bandgap wavelengths is similar at the band-edge of the perovskite.

Figures 4d–f display dOD/d*V* vs the applied potential (black lines) and the corresponding CVs (red lines). Both curves show a strong similarity, especially for the bulk CsPbBr_3_ film in DCM. This is because the change in the absorption of the CsPbBr_3_ film should be proportional to the injected charge *Q* as each injected electron leads to the cathodic dissolution of a consistent amount of CsPbBr_3_, i.e. ΔOD ∝ *Q*. Since the current *I* = d*Q*/d*t* = 1/*v*·d*Q*/d*V*, where *v* represents the scan rate in V/s, this implies that *I* should have the same potential dependence as dOD/d*V*. Hence the change in OD with potential serves as an independent measurement of cathodic dissolution of the CsPbBr_3_ material.

For the bulk film in DCM (Figure 4d), no changes in the perovskite OD are observed until −1.5 V vs Fc/Fc^+^ (potential ⓪). The CV shows charge injection when more negative potentials are applied, leading to a gradual decrease of the perovskite OD (dOD/d*V* < 0). Beyond −1.7 V vs Fc/Fc^+^ (potential ②), cathodic decomposition leads to a swift dissolution of the entire film. The observation of some cathodic decomposition in the dOD/d*V* signal beginning between potentials ⓪ and ② could be linked to the reduction of undercoordinated CsPbBr_3_ sites at the grain boundaries, which experience less electrochemical stabilization from neighboring atoms. The cathodic current and dOD/d*V* exhibit similar behavior, indicating that all injected charges are directly used for the cathodic decomposition of the bulk perovskite. This confirms the assignment of wave II as the cathodic decomposition reaction and shows that no prior side reactions take place, consistent with the absence of Pb^2+^-complexes in solution. Interestingly, the reverse scan shows a minor increase in the CsPbBr_3_ band-edge absorption (dOD/d*V* > 0) from −0.75 V vs Fc/Fc^+^, suggesting the anodic formation of CsPbBr_3_ from electrodeposited Pb^0^ and dissolved Cs^+^ and Br^–^ ions. However, the amount of CsPbBr_3_ formed during the anodic scan is considerably less than the amount lost in cathodic decomposition, in line with the irreversible character of the CV.

The measurements of the NC films in DCM (Figure 4e) and PC (Figure 4f) display similar behavior, but with important differences. In both systems, a continuous decrease of the OD (dOD/d*V* < 0) is observed even at *V*_OC_, which implies that the NCs dissolve gradually in both solvents without an applied potential. Furthermore, the reduction of Pb^2+^-complexes in solution at −1.2 V vs Fc/Fc^+^ (potential ①) leads to increased perovskite dissolution in both PC and DCM, although the effect is more pronounced in the latter case. This suggests an interplay between the NC film and the reduction of dissolved Pb^2+^-complexes. The release of free OA^–^ or Br^–^ ions from this reduction reaction may affect the solubility equilibria of the NCs, potentially promoting their dissolution. This remains speculative at present. Nonetheless, the reduction potential of the cathodic decomposition reaction is clearly reached at −1.7 V vs Fc/Fc^+^ (potential ②), as observed from the accelerated dissolution of the NCs, with dOD/d*V* becoming roughly proportional to the current.

### In Situ Spectro-electrochemical Measurement of the PL

**Figure 5:**
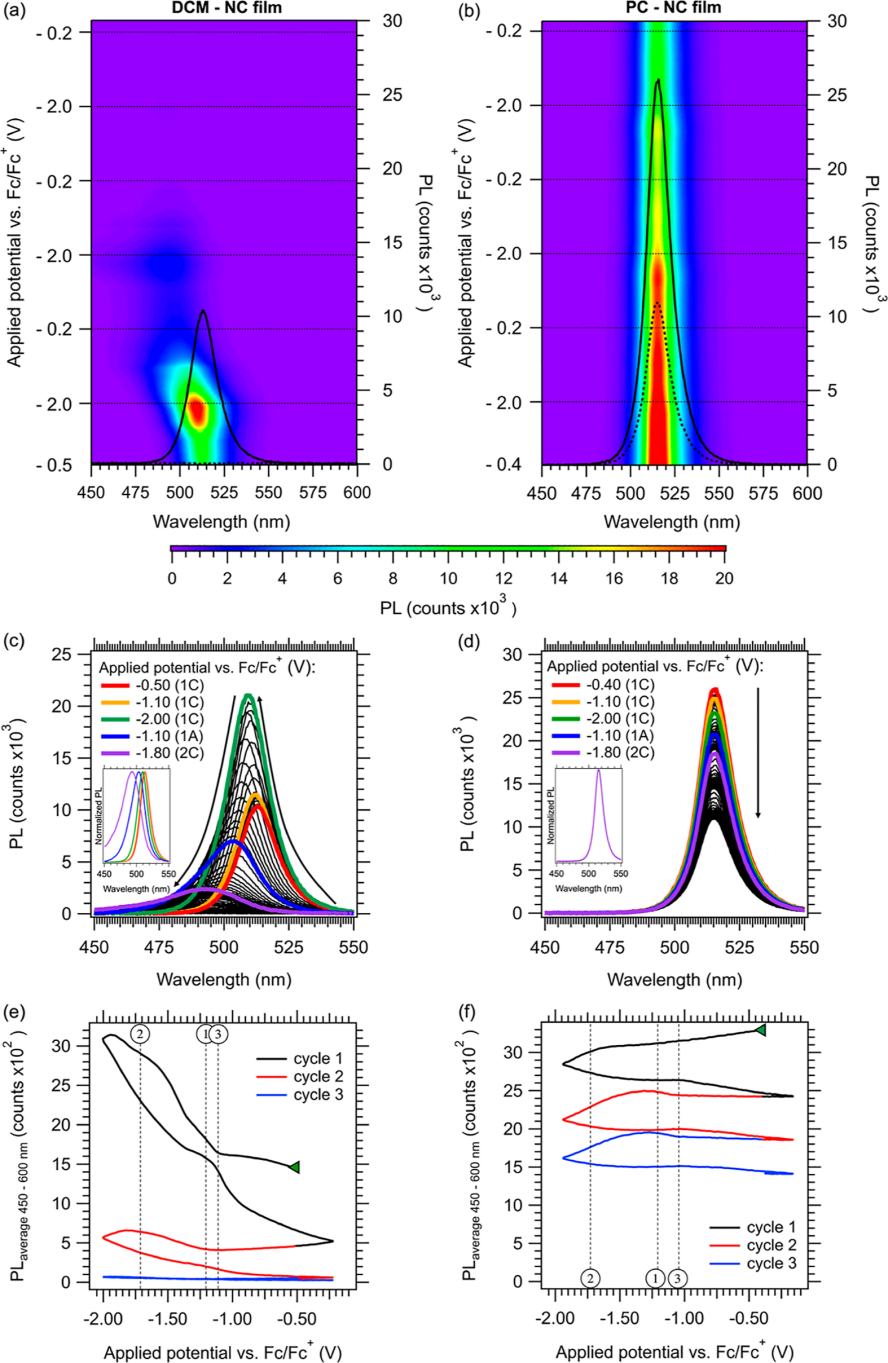
(a) False-color image of the change to the PL spectra of NC films in DCM and (b) in PC, measured in situ during the CVs shown in Figure 2a,b. The initial (solid lines) and final (dotted lines) PL spectra are overlaid on each figure. Horizontal dotted lines indicate the turning points of the CV scans. (c) PL spectra of an NC film in DCM during the CV, displaying an increase in PL intensity, along with a blueshift and broadening of the PL peak (inset). (d) PL spectra of the NC film in PC during the CV, displaying a gradual decrease of the PL intensity over time, but without a blueshift or broadening of the PL peak (inset) as observed in (c). Colored lines represent the PL spectra at specific potentials during the first cathodic cycle (1C), first anodic cycle (1A), and second cathodic cycle (2C). (e) The average PL counts of NC films in DCM and (f) in PC between 450 and 600 nm as a function of applied potential, with circled numbers indicating the potentials discussed in the text.

The differences between the electrochemical behavior of the NC films in PC and DCM are further highlighted by examining the potential-dependent changes of the PL, as shown in Figure 5. Since the bulk film shows no detectable PL, our discussion focuses on the NC films.

The NC film in DCM (Figure 5a,c,e) displays a sharp initial increase in PL intensity, coinciding with the onset of wave I at −1.1 V vs Fc/Fc^+^ (potential ③). The PL intensity roughly doubles as the potential is swept to more negative values during the first cathodic scan, and is accompanied by a blue shift and broadening of the PL peak (inset Figure 5c). This suggests that the NCs are etched due to an electrochemical surface reaction, i.e. decreasing the NC size results in an increase of the bandgap energy through quantum confinement. This is further supported by similar observed blue shifts in the OD spectra of the film during the first cycle, as shown in SI-27. The broadening of the PL peak is a result of NCs becoming more polydisperse. A swift collapse of the PL intensity is observed during the reverse scan, consistent with substantial degradation of the NCs due to cathodic decomposition in wave II.

In contrast, the PL changes of the NC film in PC (Figure 5b,d,f) show a more gradual pattern. The PL intensity exhibits a moderate decrease starting at *V*_OC_, which we attribute to the slow dissolution of the NCs, independent of the applied potential. The decline in PL intensity clearly increases as the potential is swept negative of −1.7 V vs Fc/Fc^+^ (potential ②), consistent with cathodic decomposition occurring in wave II. An increase in PL intensity is observed only in the second and third cycles, albeit to a much lesser extent. Furthermore, no blue shift or broadening of the PL peak is observed, as etched NCs will fully dissolve immediately in PC due to their high solubility. The PL retains over 40% of its original intensity after three CV cycles. The subtle PL changes are consistent with the relatively small cathodic currents and minor changes to the perovskite OD.

Altogether, these findings show that the CsPbBr_3_ NC film in PC exhibits a higher stability against cathodic decomposition compared to the films in DCM. This suggests that the electrochemical stability of CsPbBr_3_ NCs is primarily governed by surface passivation, rather than *K*_sp_ of CsPbBr_3_ in the electrolyte. We note that electrochemical measurements of NC films in MeCN, PhCN and THF yield similar results, as shown in SI-22 to SI-24. In PhCN and THF, we observe a strong initial gain in PL intensity, followed by an irreversible collapse. In contrast, the measurement in MeCN exhibits more gradual PL changes, similar to the case of PC. Overall, we note a positive correlation between the extent of the changes in PL intensity and the solubility of Pb-ligand complexes in the electrolyte (Figure 3a). This supports our notion that increasing the ligands’ surface coverage protects the NCs against cathodic decomposition.

For the NC film in DCM, the initial increase in PL intensity, the blue shift and the broadening of the PL peak all coincide with the onset of cathodic wave I (potential ③). This suggests a complex interplay between the NCs and the reduction of dissolved Pb^2+^-complexes. Potential-dependent changes of the PL intensity have been reported for various NC systems. For instance, in CdTe NCs, a strong increase in the PL quantum yield at negative potentials was attributed to the filling of trap states formed by undercoordinated Te surface atoms.^61,62^ Brovelli et al. previously observed similar PL increases of CsPbBr_3_ NC films at negative potentials and attributed the effect to a filling of static trap states.^16^ While such mechanisms cannot be ruled out, it is clear that significant electrochemical restructuring occurs during our CVs of NC films and it is likely that this is responsible for the gain in PL intensity. Since we observe that the PL increase correlates with the onset of Pb^2+^ reduction and NC etching we propose that the formation of metallic Pb^0^ domains on the electrode, or alterations in ligand surface coverage of the NCs (e.g., loss of surface Br^–^ ions which could act as trap states), can influence the electronic structure by removing trap states from the bandgap, leading to an initial increase in PLQY in the case of CsPbBr_3_ NC films in DCM.

The orthogonal solubility of CsPbBr_3_ and the Pb-ligand complexes prevents stable electrochemical measurements of CsPbBr_3_ NCs in common electrolytes. An open question remains whether an electrolyte can be found in which both the CsPbBr_3_ lattice and the surface ligand complexes exhibit a sufficiently low solubility. Hydrofluoroethers have been proposed as a promising class of electrolyte solvents for perovskite electrochemistry in literature.^47,50^ Although we measured the bulk CsPbBr_3_ solubility in methoxyperfluorobutane and found it to be very low, we did not manage to obtain electrolytes with satisfactory ionic conductivity for electrochemical measurements, i.e., without experiencing significant Ohmic overpotentials. A more promising approach for stable electrochemical measurements would be to engineer CsPbBr_3_ NCs with different surface ligands that exhibit much lower solubility in low-polarity solvents like DCM, such as zwitterionic ligands that coordinate to multiple ions on the NC surface. Alternatively, the use of core–shell CsPbBr_3_ NCs with an insoluble shell could be an interesting avenue.

## Conclusions

The results presented in this work show that the cathodic electrochemical response of CsPbBr_3_ NC films is dominated by the reduction (and oxidation) of Pb^2+^-complexes in solution. Using ICP-OES, we demonstrate that this originates from the orthogonal solubility of the CsPbBr_3_ lattice and NC surface ligand complexes in high- and low-polarity solvents. This poses an inherent challenge for electrochemical applications and measurements of CsPbBr_3_ NC films. Measurements on ligand-free CsPbBr_3_ bulk films in low-polar DCM, which are not complicated by this orthogonality, indeed show cleaner CV measurements, influenced solely by the cathodic bulk decomposition at −1.7 V vs Fc/Fc^+^. However, as we show with in situ optical measurements, NC films can be relatively stable in high-polarity solvents due to the insolubility of the ligand shell, preventing the NCs from rapid and complete degradation, and allowing for swift electrochemical measurements. When performing these measurements, we therefore, suggest a careful selection of the electrolyte solvent: low-polarity for ligand-free bulk perovskites and high-polarity for ligand-passivated NCs.

Nevertheless, cathodic decomposition occurs before the CB can be populated with electrons in all electrochemical experiments on CsPbBr_3_ NC and bulk films. This limited cathodic stability forms an inherent complication for applications that involve a semiconductor–electrolyte interface, such as in LECs or photocatalysis.

## Supporting Information Available

Experimental methods (used materials, synthesis of CsPbBr_3_ NCs, Pb(OA)_2_ and CsOA, preparation of thin film electrodes, and equipment and measurement details); characterization of CsPbBr_3_ NCs and thin films (absorbance, PL, TEM and SEM images, XRD); spectro-electrochemical measurements of CsPbBr_3_ NC electrodes in additional electrolyte solvents, or combined with supplementary characterization techniques (CV, chronoamperometry, in situ absorbance and PL measurements, XPS, XRD); solubility data of CsPbBr_3_, Pb(OA)_2_ and CsOA in a variety of solvents (ICP-OES, XRD); extended analysis of the presented spectro-electrochemical data (PDF)
