# Supplementary material for: Orthogonal Electrochemical Stability of Bulk and Surface in Lead Halide Perovskite Thin Films and Nanocrystals
Source: J Am Chem Soc. 2024 Aug 23;146(35):24415–25. doi: 10.1021/jacs.4c06340 (PMC11378294; doi:10.1021/jacs.4c06340)
Supplement: Supplementary file 1 — ja4c06340_si_001.pdf [file ja4c06340_si_001.pdf]

Supporting information for

Orthogonal Electrochemical Stability of Bulk and Surface in Lead Halide Perovskite Thin Films and Nanocrystals

Jence T. Mulder,[†,§] *Julius O.V. Monchen,*[†,§] *Yan B. Vogel,*[†] *Cheng Tai Lin,*[†] *Filippo Drago,*[‡] *Valentina M. Caselli,*[†] *Niranjan Saikumar,*[$] *Tom J. Savenije,*[†] *and Arjan J. Houtepen*[†,*]

[†] Optoelectronic Materials Section, Faculty of Applied Sciences, Delft University of Technology, Van der Maasweg 9, 2629 HZ Delft, The Netherlands
[‡] Chemistry Facility, Istituto Italiano di Tecnologia (IIT), Via Morego 30, 16163 Genova, Italy
[$] Department of Precision and Microsystems Engineering, Faculty of Mechanical Engineering, Delft University of Technology, Mekelweg 2, 2628 CD Delft, The Netherlands

Contents

SI-1 – Methods and Materials – Used Chemicals S-1
SI-2 – Methods and Materials – $CsPbBr_3$ Nanocrystal Synthesis S-2
SI-3 – Methods and Materials – $CsPbBr_3$ Nanocrystal and Bulk Film Preparation S-3
SI-4 – Methods and Materials – (Spectro-)Electrochemical Equipment and Measurement Set-up S-4
SI-5 – Methods and Materials – $CsPbBr_3$, Lead- and Cesium Oleate Synthesis – Solubility Study S-5
SI-6 – Methods and Materials – Solubility Analysis through ICP-OES Measurements S-6
SI-7 – $CsPbBr_3$ Nanocrystal Characterization S-7
SI-8 – $CsPbBr_3$ Bulk Film Characterization S-8
SI-9 – Chronoamperometry and XPS – $CsPbBr_3$ Nanocrystals – PC S-9
SI-10 – XRD on XPS Samples S-11
SI-11 – Electrochemistry – Lead Oleate – DCM S-12
SI-12 – ICP-OES Analysis of Bulk $CsPbBr_3$ Solubility – Samples S-13
SI-13 – ICP-OES Analysis of Bulk $CsPbBr_3$ Solubility – XRD S-14
SI-14 – ICP-OES Analysis of Bulk $CsPbBr_3$ Solubility – Values S-15
SI-15 – ICP-OES Analysis of Bulk $CsPbBr_3$ Solubility – Graphs S-16
SI-16 – ICP-OES Analysis of $Pb(OA)_2$ and CsOA Solubility – Values S-17
SI-17 – OD and ΔOD Spectra of $CsPbBr_3$ Films Before and After CV Measurements S-18
SI-18 – Correction to Perovskite OD in CV Measurements S-19
SI-19 – Correlation of Injected Charge to Change of OD for NC Samples S-20
SI-20 – Correlation of the PL-peak Position to the Applied Potential and Time S-21
SI-21 – Electrochemistry – Lead Bromide – PC S-22
SI-22 – $CsPbBr_3$ Nanocrystal Electrochemistry – MeCN S-23
SI-23 – $CsPbBr_3$ Nanocrystal Electrochemistry – PhCN S-24
SI-24 – $CsPbBr_3$ Nanocrystal Electrochemistry – THF S-25
SI-25 – Electrochemistry – Background Current in Blank CVs of the Used Electrolytes S-26
SI-26 – Stability of $CsPbBr_3$ Nanocrystal Thin Films S-27
SI-27 – OD Spectra of $CsPbBr_3$ Films at Different Potentials during CV Measurements S-28
References S-29

SI-1 – Methods and Materials – Used Chemicals

Nanocrystal synthesis and film preparation

Lead acetate trihydrate ($Pb(CH_3COO)_2 \cdot 3H_2O$, 99.999% trace metal basis), benzoyl bromide (97%), 1-octadecene (ODE, technical grade, 90%), toluene (anhydrous, 99.8%), methyl acetate (anhydrous, 99.5%), mercaptopropionic acid (MPA, ≥99.0%), 1,8-octane dithiol (8DT, ≥97%) were purchased from Sigma-Aldrich. Oleic acid (OA, extra pure) was purchased from Fisher Scientific. Cesium carbonate (Cs_2CO_3, Puratronic, 99.994%) was purchased from Alfa Aesar. Oleylamine (OLA, 80−90% C18, ≥96.0% (primary amine)) was purchased from Acros Organics. Methanol (anhydrous, max 0.003% H_2O, Seccosolv) was purchased from VWR international. ITO conductive substrates (20 Ohms/sq) were purchased from PGO GmbH.

Bulk film preparation

Cesium bromide (CsBr, 99.999% perovskite grade) and lead bromide ($PbBr_2$, 98+%) were purchased from Sigma-Aldrich. ITO conductive substrates (20 Ohms/sq) were purchased from PGO GmbH.

Electrochemistry

Propylene carbonate (PC, anhydrous, 99.7%), dichloromethane (DCM, anhydrous, ≥99.8%), tetrahydrofuran (THF, anhydrous, ≥99.9%), acetonitrile (MeCN, anhydrous, 99.8%), benzonitrile (PhCN, anhydrous, ≥99%), tetrabutylammonium hexafluorophosphate ($TBAPF_6$, for electrochemical analysis, ≥99.0%), tetrabutylammonium bromide (TBABr, ≥98.0%), ferrocene (98%) were purchased from Sigma-Aldrich.

ICP measurements

Propylene carbonate (PC, anhydrous, 99.7%), dichloromethane (DCM, anhydrous, ≥99.8%), tetrahydrofuran (THF, anhydrous, ≥99.9%), acetonitrile (MeCN, anhydrous, 99.8%), benzonitrile (PhCN, anhydrous, ≥99%), N,N-dimethylformamide (DMF, anhydrous, 99.8%), methyl acetate (anhydrous, 99.5%), ethylene carbonate (anhydrous, 99%), chloroform (anhydrous, 99%), tetrachloroethene (anhydrous, ≥99.0%), methoxy perfluorobutane (HFE 7100, 99%, mixture of n- and iso-butyl isomers), dimethyl sulfoxide (DMSO, anhydrous, ≥99.9%), N-methylpyrrolidone (NMP, anhydrous, ≥99.5%), isopropanol (anhydrous, 99.5%), chlorobenzene (anhydrous, 99.8%), 1,2-dichlorobenzene (anhydrous, 99%), benzene (anhydrous, 99.8%), ethyl acetate (anhydrous, 99.8%), pyridine (anhydrous, 99.8%) diethyl carbonate (anhydrous, 99%), cesium bromide (CsBr, 99.999% perovskite grade) and lead bromide ($PbBr_2$, 98+%) were purchased from Sigma-Aldrich. Milli-Q water was obtained from a Milli-Q Advantage A10 system (Merck Millipore, 18.2 MΩ·cm, 2 ppb TOC). Methanol (anhydrous, max 0.003% H_2O, Seccosolv) was purchased from VWR international. Toluene (anhydrous, ≥99.8%), acetone (anhydrous, ≥99.8%) and ethanol (anhydrous, ≥99.8%) were purchased from VWR Chemicals. Hexane (anhydrous, >96.0%) was purchased from TCI.

Lead oleate

Lead(II) oxide (PbO, 99.999%), acetonitrile (MeCN, ≥99.9% Chromasolv), isopropanol (≥99.5%), triethylamine (≥99% for synthesis) and methanol (≥99.8%) were purchased from Sigma-Aldrich. Oleic acid (OA, extra pure), trifluoroacetic acid (HTFA, ≥99.0%, for HPLC), trifluoroacetic anhydride (TFAA, ≥99.0%) were purchased from Fisher Scientific.

Cesium oleate

Cesium carbonate (Cs_2CO_3, Puratronic, 99.994%) was purchased from Alfa Aesar. Oleic acid (OA, extra pure) was purchased from Fisher Scientific. Methanol (≥99.8%) was purchased from Sigma-Aldrich.

SI-2 – Methods and Materials – $CsPbBr_3$ Nanocrystal Synthesis

Nanocrystal precursor preparation

A stock solution of metal precursors was prepared by combining 760 mg of $Pb(CH_3COO)_2{\cdot}3H_2O$ and 160 mg of Cs_2CO_3 together with 50 mL ODE, 10 mL OLA and 3 mL OA in a three-necked round bottom flask. This mixture was attached to a Schlenk line and flushed 3 times with dry nitrogen. Whilst stirring, the mixture was then heated to 120°C for one hour under a vacuum pressure of <0.2 mbar, to remove any water, oxygen and other volatile chemical species from the reaction mixture. The precursor was subsequently cooled down to room temperature, transferred to nitrogen filled vials and stored in a nitrogen filled glovebox.

$CsPbBr_3$ nanocrystal synthesis

The following synthesis protocol has been adapted from Imran *et al.*[1]

6.3 mL of the metal/ligand precursor solution was added to a nitrogen filled 25 mL three-necked flask with a thermocouple insert, attached to a Schlenk line. The mixture was quickly heated to 185°C, and under vigorous stirring 71 µL of benzoyl bromide diluted in 500 µL degassed ODE was swiftly injected into the metal precursor mixture. The mixture directly turned yellow and after exactly 10 seconds, the heating mantle was removed, and the flask was quickly cooled down using an air gun. Once the reaction mixture reached room temperature, 2 mL of anhydrous toluene was added, and the contents were subsequently transferred to nitrogen filled vials. The crude mixture was centrifuged at 3800 rpm for 10 minutes to separate the NCs from the solution. After centrifugation, the supernatant was discarded in a glove box, and the solid pellet was redispersed in 2 mL of anhydrous toluene.

To remove most of the residual precursors and solvents, the NC solution was washed by adding 2 mL of methyl acetate to destabilize the dispersion. The cloudy solution was centrifuged at 6000 rpm for 10 minutes and after discarding the supernatant, the NC pellet was redispersed in 4 mL of anhydrous toluene. The dispersion was then passed through a 0.2 µm PTFE syringe filter, and the filtrate was stored inside a nitrogen filled glovebox.

An overview of the obtained sample characteristics is provided in SI-7.

SI-3 – Methods and Materials – $CsPbBr_3$ Nanocrystal and Bulk Film Preparation

Nanocrystal thin film preparation

ITO conductive substrates were cleaned by rinsing them thoroughly with isopropanol. The rinsed substrates were dried using an airgun and then further treated in a Bioforce ProCleaner UV/Ozone cleaner for 1 hour. Subsequently, the substrates were submerged in a methanolic solution of 1 vol% MPA for at least 24 hours, to functionalize the oxide layer with surface thiol groups. Once functionalized, the substrates were rinsed with clean methanol to remove excess MPA. 200 µL of NC solution was drop-cast on the functionalized, dry substrates and the toluene was evaporated over time without application of a vacuum or heating. To cross-link the NCs, the dry films were submerged in a 0.1 M 8DT solution in methyl acetate. The films were then dipped in clean methyl acetate to remove residual ligands. To counter NC degradation, residual solvents were directly removed by putting the films under vacuum. The films were thereafter stored in a nitrogen-filled glovebox until use.

An overview of the obtained sample characteristics is provided in SI-7.

Bulk thin film preparation

ITO conductive substrates were cleaned by rinsing them thoroughly with isopropanol. The rinsed substrates were dried using an airgun and then further treated in a Bioforce ProCleaner UV/Ozone cleaner for 1 hour. Subsequently, the substrates were mounted inside an AJA ATC Orion 4 thermal evaporator. Layer by layer, 50 Å CsBr (source heated to 394 °C) and 44 Å $PbBr_2$ (source heated to 272 °C) were alternately deposited at an evaporation rate of 0.3 Å/s. After a total of 100 cycles (50 cycles per material), the evaporation was stopped and the films were brought into a nitrogen filled glovebox. Here, the films were thermally annealed on a hot plate at 100 °C for 1 hour.

An overview of the obtained film characteristics is provided in SI-8.

SI-4 – Methods and Materials – (Spectro-)Electrochemical Equipment and Measurement Set-up

All (spectro-)electrochemical measurements were performed in a nitrogen-filled glovebox with H_2O and O_2 levels of respectively ≤0.5 ppm and ≤0.1 ppm. Measurements were performed with an Autolab PGSTAT128N potentiostat with a bipotentiostat BA module. The electrochemical cell consisted of a 1 × 2 × 4 cm quartz cuvette, containing approximately 6 mL of electrolyte (*e.g.*, 0.1 M $TBAPF_6$ in PC) in which the NC working electrode (WE), a reference electrode (RE) and a Pt sheet as a counter electrode (CE) were submerged. In general, the CVs were repeated three times with a scan rate of 10 mV/s.

An Ag wire was used as a pseudo-reference electrode for electrochemical measurements in PC. For the electrochemical measurements in DCM, ACN, THF an PhCN, a leakless miniature Ag/AgCl reference electrode (ET072 from EDAQ) was used. In all cases, the potential of the reference electrode was calibrated with a ferrocene couple directly after each measurement.

Potential-dependent changes in the optical density (OD) and photoluminescence (PL) of the films were recorded with a fiber-coupled UV−vis spectrometer (Ocean Optics USB2000), using an Ocean Optics DH 2000 light source and a 405 nm laser diode respectively.

SI-5 – Methods and Materials – $CsPbBr_3$, Lead- and Cesium Oleate Synthesis – Solubility Study

$CsPbBr_3$ Synthesis

Equimolar amounts of CsBr and $PbBr_2$ were added in excess to 26 different solvents (listed in SI-14) to prepare saturated solutions of $CsPbBr_3$. The white precursors slowly converted to an orange precipitate in most solvents (see SI-12), indicating the formation of $CsPbBr_3$, as confirmed by XRD (see SI-13).

Lead oleate synthesis

Lead oleate ($Pb(OA)_2$) was synthesized following a procedure of Hendricks et al.[2]

In short, 10 g of lead(II) oxide and 20 mL of acetonitrile were added to a 100 mL flask, which was then cooled in an ice bath. Subsequently, 0.7 mL trifluoroacetic acid and 6.2 mL trifluoroacetic anhydride were added and the mixture was stirred until all solids were completely dissolved. This solution was then added to a 500 mL Erlenmeyer flask containing a mixture of 25 g of oleic acid, 180 mL isopropanol and 10 g of triethylamine, resulting in the formation of a white precipitate. This precipitate was completely dissolved by heating, up to the point the solution refluxes slowly. Once the solution turned clear, the heating was switched off and the solution was left to cool back to room temperature over time. Subsequently, the solution was stored in the freezer for a day to precipitate the lead oleate out of solution.

To remove any side products and unreacted precursors, the $Pb(OA)_2$ was washed 3 times over a glass filter with 300 mL of methanol whilst stirring the slurry with a glass rod. The solids were dried under vacuum for a day in order to remove all volatile solvents, and the dried $Pb(OA)_2$ was subsequently stored in a nitrogen filled glovebox.

Cesium oleate synthesis

Cesium oleate (CsOA) was synthesized following a procedure of Bohn et al.[3]

In short, 33 mg of Cs_2CO_3 and 10 mL of oleic acid were added to a 25 mL three necked flask. The contents were then heated to 100 °C and kept at this temperature until all Cs_2CO_3 fully reacted away. The solution was subsequently cooled down to room temperature and then centrifuged to collect all the precipitated cesium oleate. The liquid fraction was then decanted and the solids were washed 3 times with 50 mL of methanol and subsequently centrifuged again to remove all side products and unreacted precursors. The solids were then dried under vacuum for a day to remove all solvents, and stored in a nitrogen filled glovebox.

SI-6 – Methods and Materials – Solubility Analysis through ICP-OES Measurements

The solubility of bulk $CsPbBr_3$, $Pb(OA)_2$ and CsOA in various solvents was determined through inductively coupled plasma – optical emission spectroscopy (ICP-OES), using saturated solutions of the respective analytes.

Filtration and evaporation of the saturated solutions

Saturated solutions of $CsPbBr_3$, $Pb(OA)_2$ and CsOA were prepared by adding an excess of the respective analyte, followed by filtration over a 0.2 µm PTFE syringe filter to remove any precipitate. Samples of the resulting clear saturated solutions were transferred to vials and evaporated on a hot plate under a vacuum of <1 mbar, leaving the solid residues behind. The sample mass of each saturated solution was adjusted based on the estimated solubility of the analyte in the solvent, ensuring that the analyte concentration in the final aqueous samples is within the calibration range for ICP-OES.

ICP-OES sample preparation and analysis

The samples containing $CsPbBr_3$ solid residue were each digested in 2 mL aqua regia. 1 mL of the solution was diluted with Milli-Q water in a volumetric flask to a total volume of 10 mL to analyze the Pb concentration. To determine the elemental Cs concentration, 1 mL of the solution was diluted with a lithium-buffer in a volumetric flask to a total volume of 10 mL.

The samples containing $Pb(OA)_2$ or CsOA were each digested in 1 mL of aqua regia. The resulting solutions were diluted with Milli-Q water and a lithium-buffer respectively, following the same procedure described above.

ICP-OES was conducted using the spectral lines at 220 nm and 217 nm to measure the Pb concentration, and 455 nm and 672 nm for the Cs concentration. The ICP-OES instrument was calibrated for elemental Pb concentrations ranging from 0.005 to 20 ppm and Cs concentrations from 20 to 200 ppm. Practical detection limits for Pb and Cs are taken as 0.05 ppm and 20 ppm respectively, based on measurements of blank control samples and the standard deviation of the measurements.

ICP-OES provides the elemental concentrations in the measured aqueous dilutions. The corresponding molar saturation concentrations in the evaporated solutions were calculated via equation 1.

$$C_{element}^{organic}\left(\frac{\mathrm{mmol}}{\mathrm{L}}\right) = \frac{\mathrm{C}_{\mathrm{element}}^{\mathrm{aqueous}}\left(\frac{\mathrm{mg}}{\mathrm{kg}}\right)\cdot \mathrm{M}^{\mathrm{aqueous}}\,(\mathrm{kg})}{MW_{element}\left(\frac{\mathrm{mg}}{\mathrm{mmol}}\right)\cdot \mathrm{V}^{\mathrm{organic}}\,(\mathrm{L})} \tag{1}$$

Here, $C_{element}^{aqueous}$ represents the elemental concentration in the aqueous sample measured by ICP-OES, $M^{aqueous}$ is the total mass of the aqueous sample, $MW_{element}$ is the molecular weight of the probed element, $V^{organic}$ is the volume of the saturated solution that was evaporated, and $C_{element}^{organic}$ is the molar saturation concentration in that sample.

SI-7 – $CsPbBr_3$ Nanocrystal Characterization

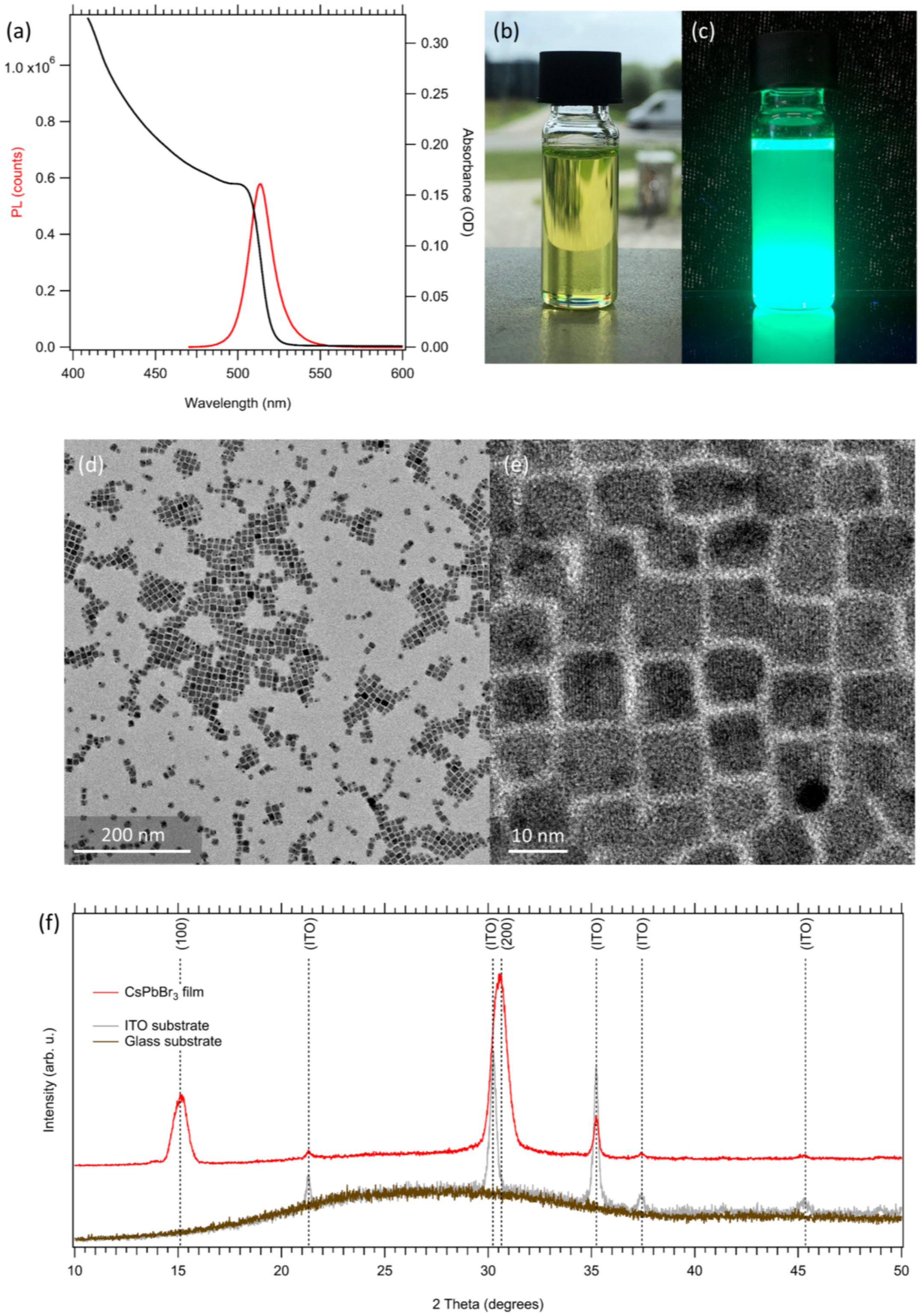

Figure S-1. (a) Absorbance and emission spectra of $CsPbBr_3$ NCs dispersed in toluene. (b) Photographs of a $CsPbBr_3$ NC sample under ambient and (c) UV illumination. (d) Large area and (e) small area TEM images of the $CsPbBr_3$ NCs, showcasing the uniformity and crystallinity of the material. (f) An XRD diffractogram of a cubic $CsPbBr_3$ NC film, indicating a phase-pure sample, unaffected by the film formation procedure.

SI-8 – $CsPbBr_3$ Bulk Film Characterization

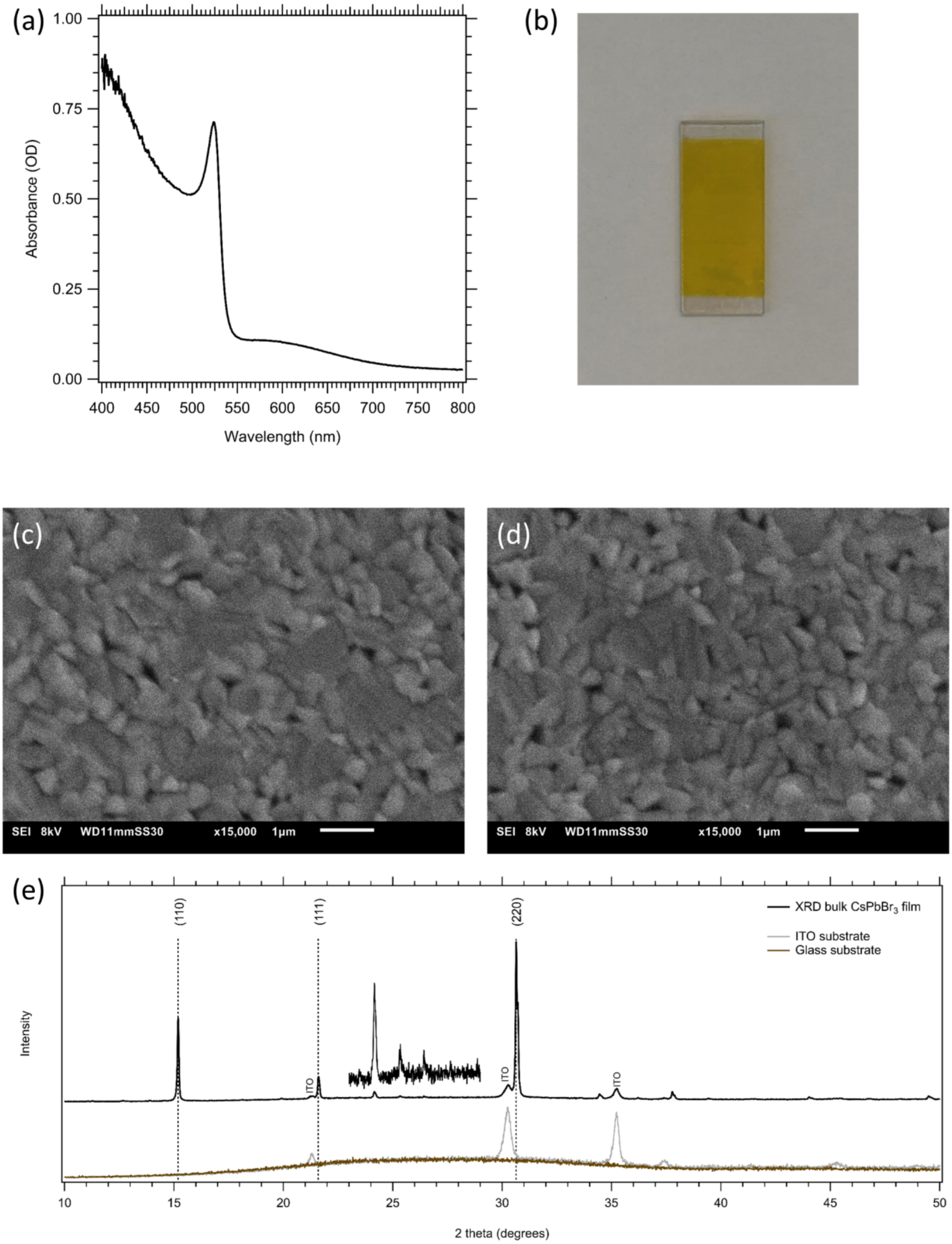

Figure S-2. (a) Absorbance spectrum of an evaporated $CsPbBr_3$ bulk film, pictured in (b). (c, d) SEM images of an evaporated $CsPbBr_3$ film, showcasing the large $CsPbBr_3$ grains. (e) An XRD diffractogram, showing that the deposited material is orthorhombic $CsPbBr_3$, best identified through the additional reflections between 24 and 28 degrees (see inset).

SI-9 – Chronoamperometry and XPS – $CsPbBr_3$ Nanocrystals – PC

Figure S-3 and S-4 show chronoamperograms and XPS-depth profiles respectively of four different $CsPbBr_3$ NC thin films after applying different potentials for 120 seconds. The chronoamperograms exhibit a potential-dependent response of the cathodic current due to the required overpotential for the formation of Pb^0 clusters. For the film held at -0.95 V, the cathodic current reaches a maximum after approximately 30 seconds as the applied overpotential for nucleation is small. Conversely, the films held at -1.30 V and -1.80 V show a much faster current rise due to the larger applied overpotential. When a film scanned for three CV cycles (between -1.80 V and 0 V) was subsequently held at 0 V for 120 seconds, the injected positive charge is small and metallic Pb^0 remains clearly visible through XPS. This shows that the Pb^0 clusters are not fully oxidized after 120 seconds at the anodic potential, explaining why the nucleation overpotential appears only in the first CV cycle.

The Pb4f XPS-scan shows two signals from the Pb4f7 and Pb4f5 with two distinct peaks for each of them. The peaks at lower binding energy correspond to metallic Pb^0, whereas the peaks at higher binding energy are attributed to Pb^{2+} in the $CsPbBr_3$ phase. The depth-profiles of the films held at -0.95 V, -1.30 V and -1.80 V show that the amount of reduced Pb^0 increases at more negative potentials.

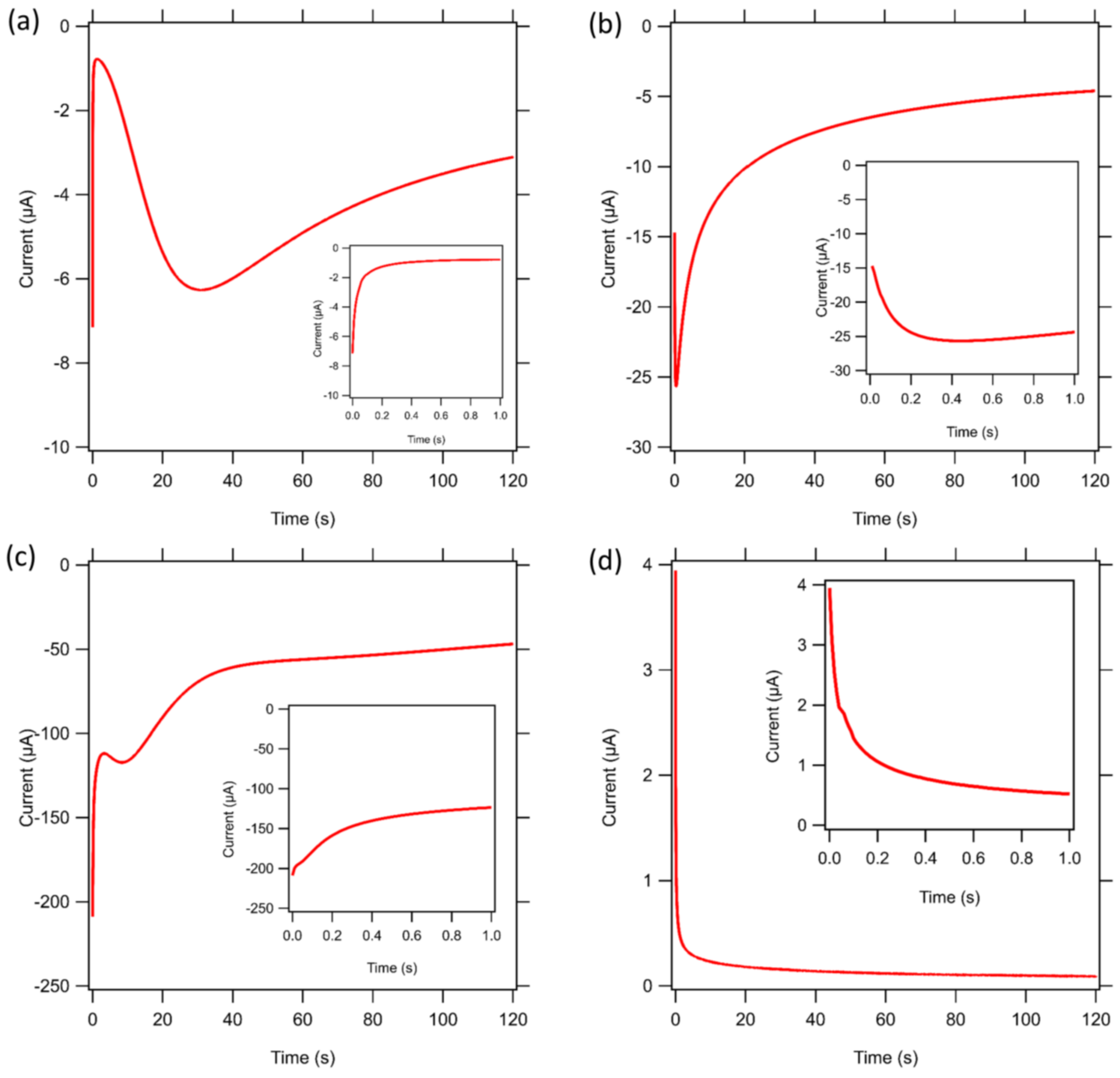

Figure S-3. Chronoamperograms of NC films held for 120 seconds at (a) -0.95 V, (b) -1.30 V, (c) -1.80 V, and (d) a NC film after three CV cycles and subsequently held at 0 V *versus* Fc/Fc^+.

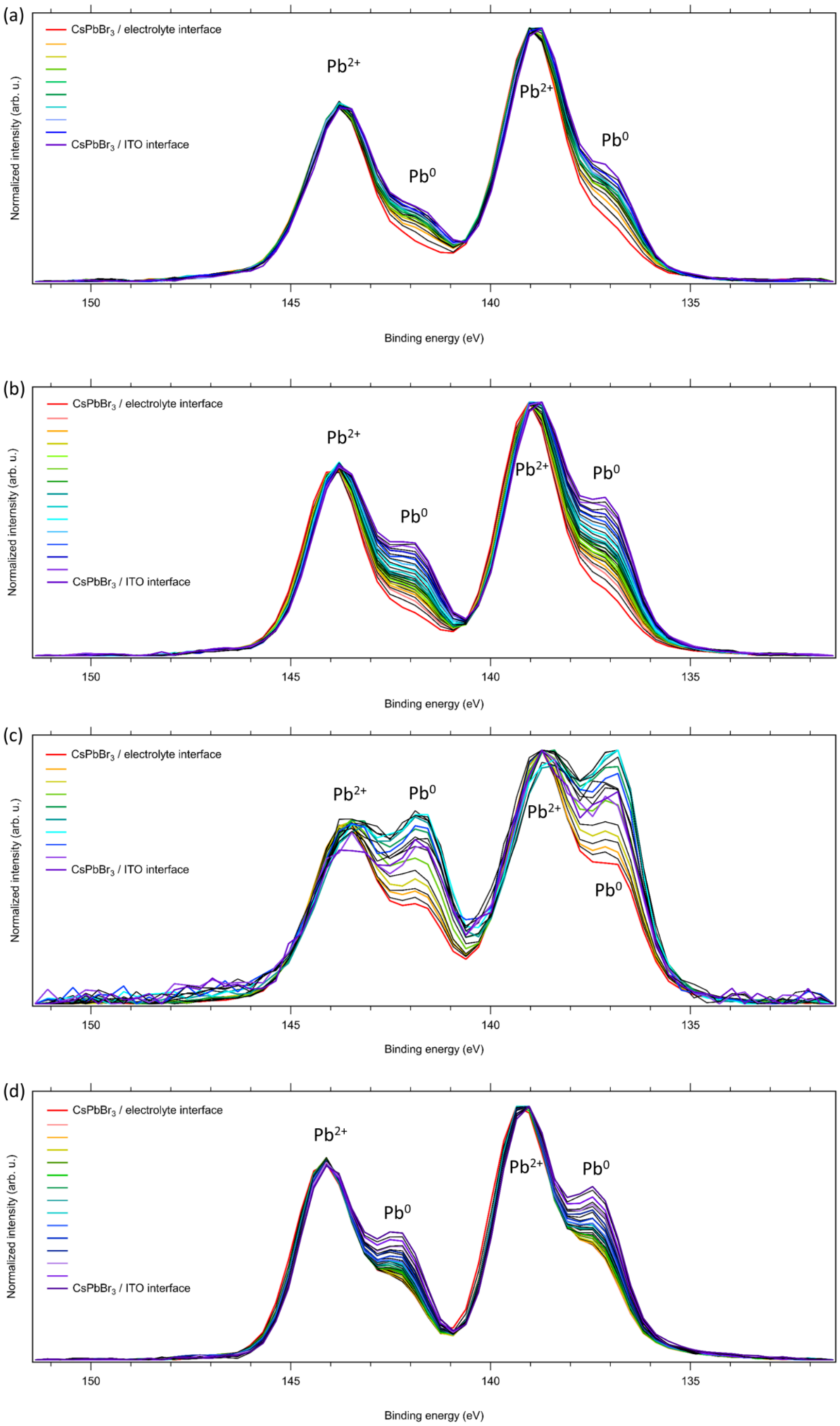

Figure S-4. XPS-depth profiles of NC films held for 120 seconds at (a) -0.95 V, (b) -1.30 V, (c) -1.80 V, and (d) a NC film after three CV cycles and subsequently held at 0 V *versus* Fc/Fc^+.

SI-10 – XRD on XPS Samples

Figure S-5 displays XRD diffractograms of NC films that were held at different potentials in PC and DCM. The films held at 0 V, -0.95 V, -1.30 V, and -1.80 V *versus* Fc/Fc^+ in PC correspond to same films used for XPS measurements and chronoamperometry (SI-9). The diffractograms show minimal changes after applying different cathodic potentials. From this we infer that the formation of metallic Pb^0 clusters on $CsPbBr_3$ NC films is difficult to detect through XRD. Compared to a pristine film that has been held at the open circuit potential (OCP) in the electrolyte (red), no significant differences are observed, except for a small signal of metallic Pb^0 for the film held at -1.80 V *versus* Fc/Fc^+. A film that was subjected to -1.80 V in DCM (black) shows stronger signals for Pb^0, as more significant cathodic decomposition of the $CsPbBr_3$ NCs has taken place.

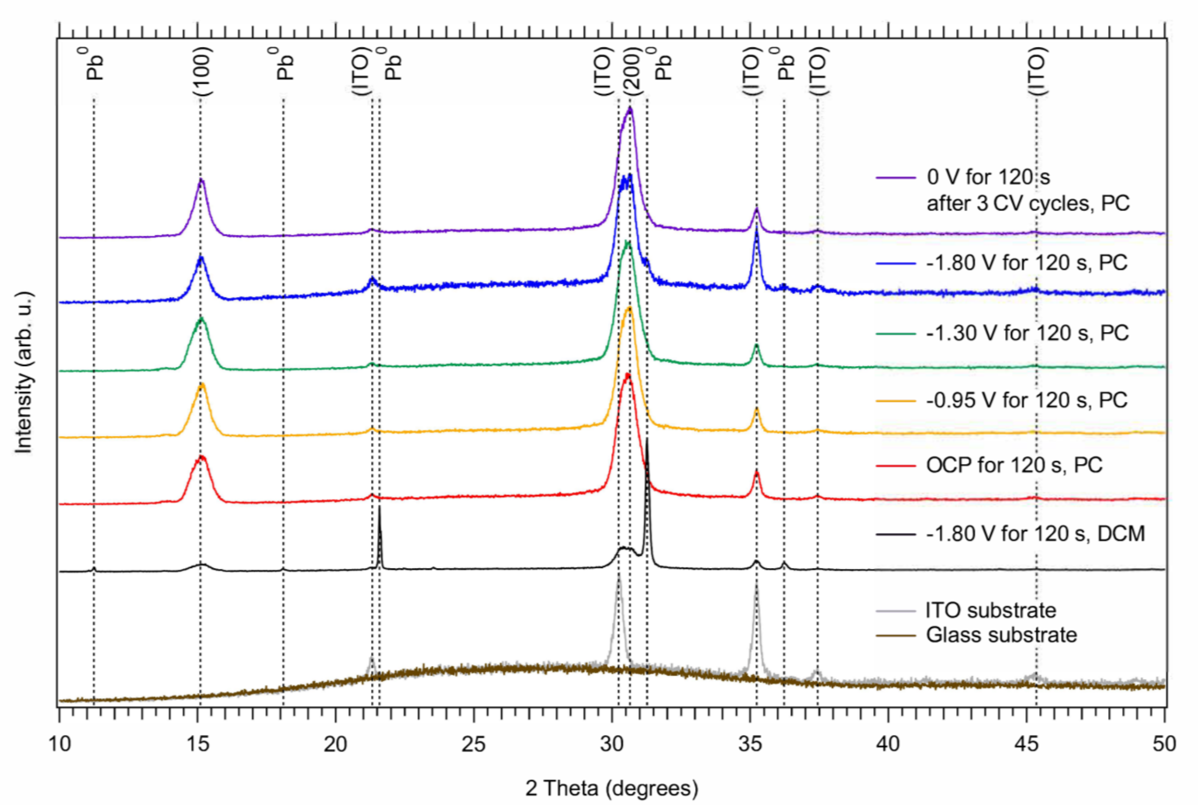

Figure S-5. XRD diffractograms of NC films held for 120 seconds at -0.95 V in PC, -1.30 V in PC, -1.80 V in PC and DCM, open circuit potential (OCP) in PC, and a NC film after three CV cycles and subsequently held at 0 V in PC. All potentials are referenced *versus* Fc/Fc^+.

SI-11 – Electrochemistry – Lead Oleate – DCM

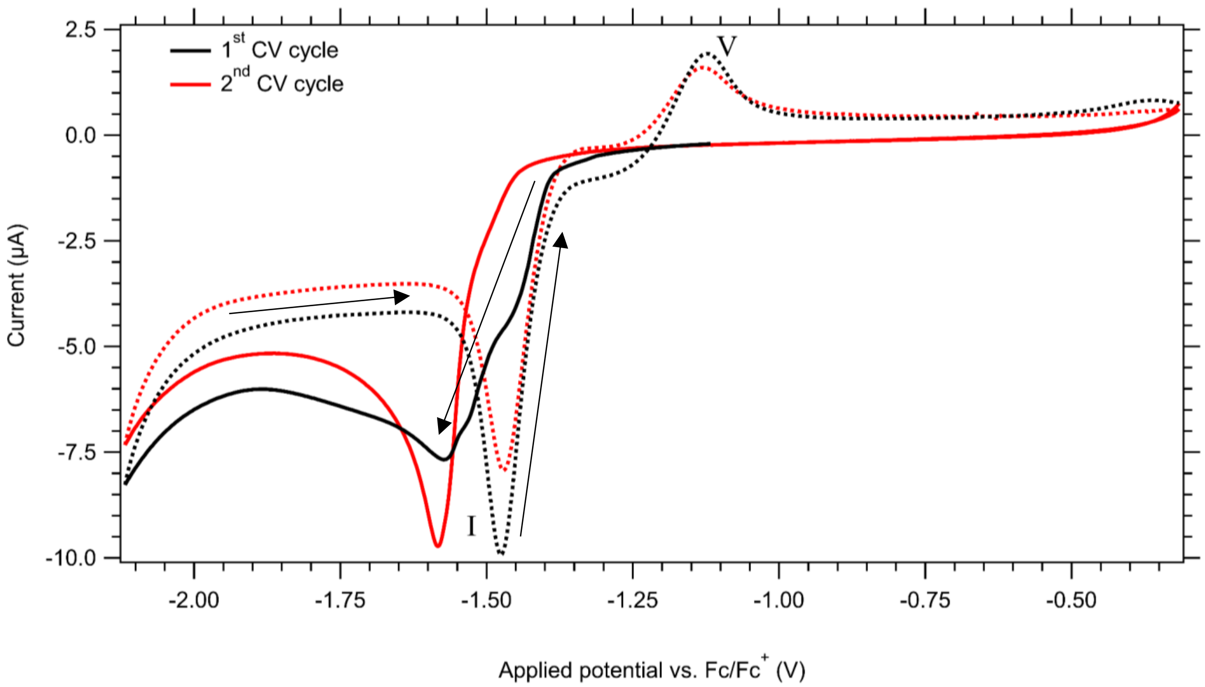

Figure S-6. A CV of an undersaturated solution of $Pb(OA)_2$ in DCM with 0.1 M $TBAPF_6$ supporting electrolyte, using a scan rate of 10 mV/s.

From the CV of $Pb(OA)_2$ in DCM (Figure S-6) we infer that Pb^{2+} ions, complexed to oleate ligands to facilitate their dissolution, exhibit a reduction potential of approximately -1.4 V *versus* Fc/Fc^+ in DCM. The potential of the reduction wave is similar to the onset potential of the first cathodic wave for the NC film in DCM, as discussed in the main text. We tentatively attribute the cathodic wave (I) and anodic wave (V) to the reduction and re-oxidation of Pb^{2+} ions complexed to oleate ligands.

I: $Pb(OA)_2 + 2\ e^- \longrightarrow Pb^0 + 2\ OA^-$

V: $Pb^0 + 2\ OA^- \longrightarrow Pb(OA)_2 + 2\ e^-$

Interestingly, the CV in Figure S-6 shows not only a reduction peak in the negative scan direction but also one in the positive scan direction. This effect persists reversibly across the second (and subsequent) cycles, suggesting it is unrelated to the initial formation of lead metal clusters or the decomposition of $Pb(OA)_2$. The electrochemical feature remains unexplained, requiring further investigation to elucidate the underlying mechanism.

SI-12 – ICP-OES Analysis of Bulk $CsPbBr_3$ Solubility – Samples

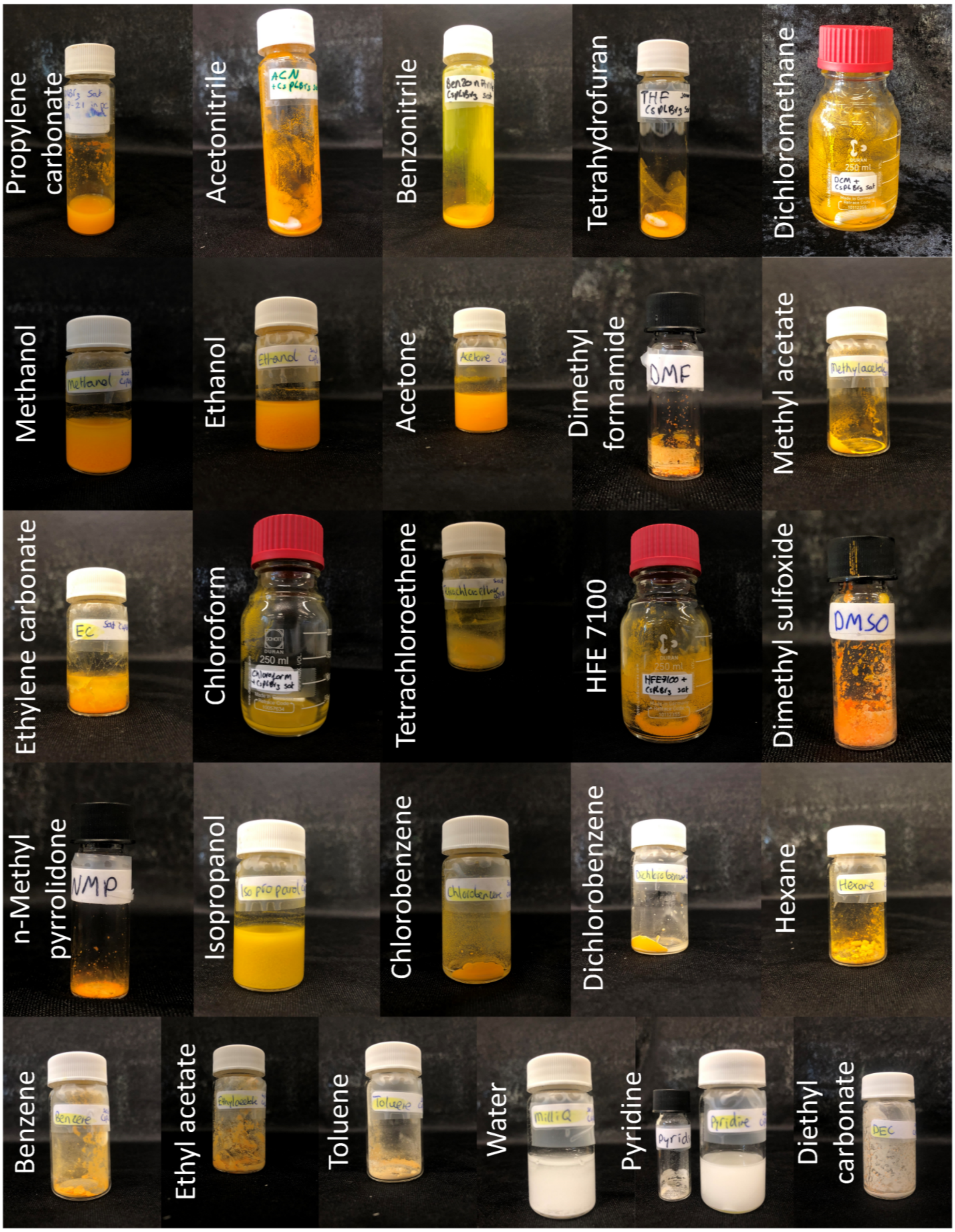

Figure S-7. Photographs of the precipitates from the saturated solutions used for the determination of the solubility product (K_{sp}) of bulk $CsPbBr_3$. The orange color indicates the formation of $CsPbBr_3$ from the white precursors, CsBr and $PbBr_2$, which were added in excess to the solvents. This demonstrates that $CsPbBr_3$ has a lower solubility product in these solvents than the precursors, so that the equilibrium concentration of the ions in the supernatant reflects the solubility product K_{sp} of $CsPbBr_3$, as required for these measurements.

SI-13 – ICP-OES Analysis of Bulk $CsPbBr_3$ Solubility – XRD

To confirm that $CsPbBr_3$ is indeed the most stable crystal phase with the lowest solubility product, XRD was performed on the orange precipitate that formed, as well as on two samples where the precipitate remained white (water and pyridine). As depicted in Figure S-8, all four orange samples show identical diffraction patterns, corresponding to practically phase-pure orthorhombic $CsPbBr_3$. This strengthens the conclusion that the ICP-OES measurements (SI-14) represent the bulk solubility product of $CsPbBr_3$ in the respective solvents. Conversely, the white precipitates in water and pyridine show XRD reflections that do not correspond to $CsPbBr_3$, nor to CsBr and $PbBr_2$. This indicates that another material composition has the lowest solubility, resulting in a different Cs:Pb ratio in solution. Interestingly, when the water evaporated from the white dispersion, orange $CsPbBr_3$ appeared (Figure S-9b). From these observations, we infer that as long as the precipitate wetted by the solvent is orange, we measure the bulk solubility product of $CsPbBr_3$.

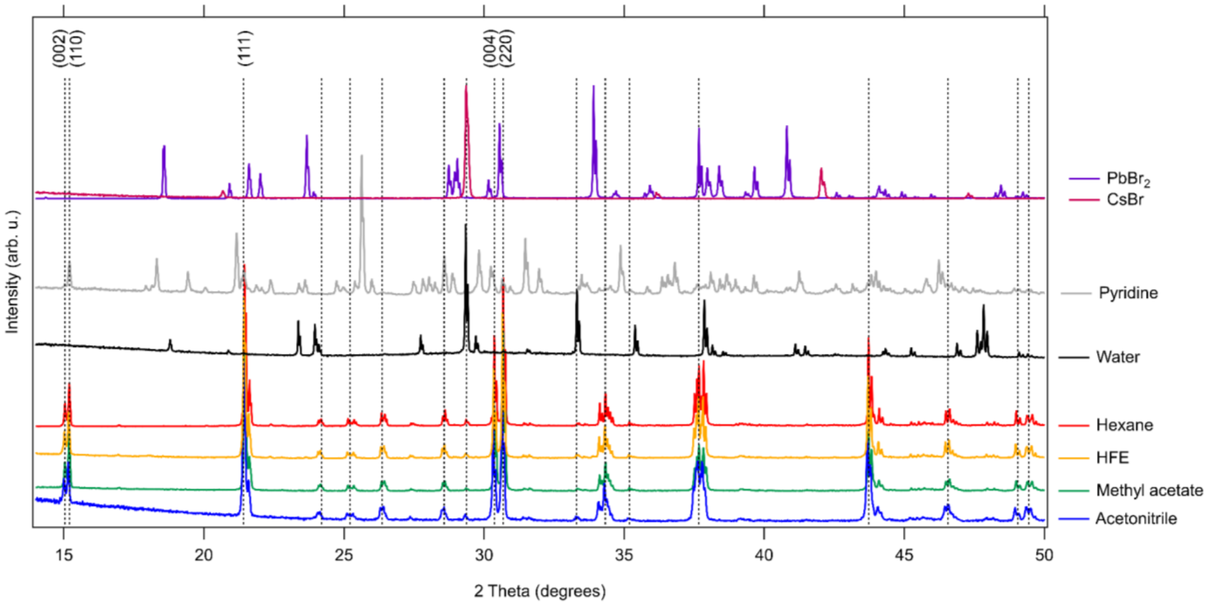

Figure S-8. XRD diffractograms of various samples shown in Figure S-7, demonstrating that the orange precipitates correspond to phase-pure orthorhombic $CsPbBr_3$. The white precipitates do not correspond to $CsPbBr_3$, nor do they correspond to the $PbBr_2$ or CsBr precursors used, suggesting the precipitation of a different material.

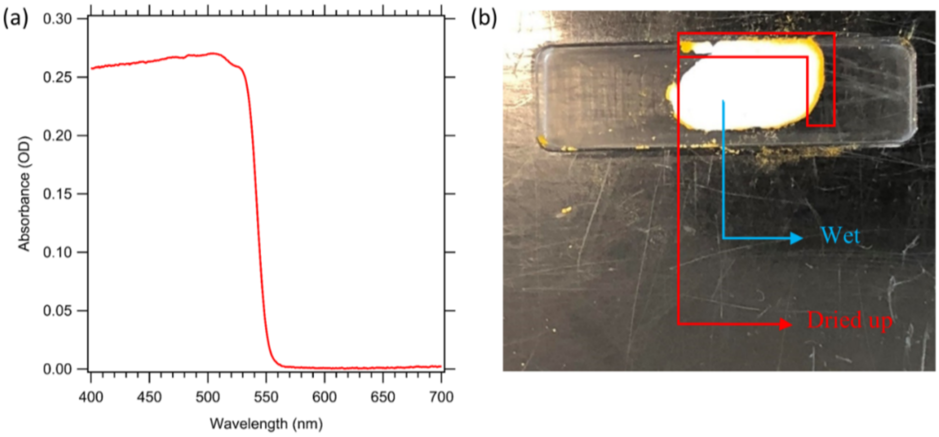

Figure S-9. (a) The absorbance spectrum of the orange $CsPbBr_3$ powder, obtained from adding equimolar amounts of CsBr and $PbBr_2$ to hexane. (b) A picture of the white precipitate in water, showing the formation of orange $CsPbBr_3$ once the water evaporates from the solids.

SI-14 – ICP-OES Analysis of Bulk $CsPbBr_3$ Solubility – Values

Table S-1. The saturation concentration of $CsPbBr_3$ in a number of solvents, measured through ICP-OES analysis of Pb. ICP-OES measurements of Cs were disregarded for further analysis due to the higher detection limit for Cs (20 ppm) than for Pb (0.05 ppm). The solubility product was determined from the measured Pb concentration, using $K_{sp,CsPbBr_3} = [Cs^+][Pb^{2+}][Br^-]^3 = 27\,[Pb^{2+}]^5$ as an approximation. As discussed in the main text, the expected electrochemical stabilization of $\mathrm{E}^{0'}_{\mathrm{CsPbBr_3/Pb}}$ *versus* $\mathrm{E}^{0'}_{Pb^{2+}/\mathrm{Pb}}$ is determined by $\frac{\mathrm{RT}}{2\mathrm{F}}\ln(\mathrm{K_{sp}})$. For the samples where Pb or Cs concentrations were below the detection limit, the values corresponding to the detection limit concentration are indicated (*without* bold formatting). In these cases, the actual K_{sp} values could be equal to or lower than the reported values, resulting in electrochemical stabilization potentials that are equal to or more negative than the reported potentials.

	Solvent	Volume used (mL)	[Pb] from ICP (ppm)	Saturation [Pb] (mM)	Calculated $K_{sp,CsPbBr3}$ (mol^5/L^5)	**Expected stabilization vs. $E^{0'}_{Pb^{2+}/Pb}$ (V)**	[Cs] from ICP (ppm)	Saturation [Cs] (mM)
1	Propylene carbonate	1.99	**49.08**	**2.38E+00**	**2.07E-12**	**-0.35**	≤20	≤1.5E+00
2	Dichloro-methane	238.12	≤0.05	≤2.03E-05	≤9.23E-38	≤ -1.09	≤20	≤1.3E-02
3	Acetonitrile	24.61	**13.50**	**5.30E-02**	**1.13E-20**	**-0.59**	**22**	**1.3E-01**
4	Benzonitrile	19.75	**5.19**	**2.54E-02**	**2.84E-22**	**-0.64**	≤20	≤1.5E-01
5	Tetrahydrofuran	29.96	**2.69**	**8.67E-03**	**1.32E-24**	**-0.71**	≤20	≤1.0E-01
6	Methanol	1.00	≤0.05	≤4.83E-03	≤7.07E-26	≤ -0.74	≤20	≤3.0E+01
7	Ethanol	1.00	≤0.05	≤4.83E-03	≤7.07E-26	≤ -0.74	≤20	≤3.0E+00
8	Acetone	1.00	**2.83**	**2.73E-01**	**4.13E-17**	**-0.48**	≤20	≤3.0E+00
9	Dimethyl-formamide	0.05	**45.33**	**8.75E+01**	**1.39E-04**	**-0.11**	≤20	≤6.0E+01
10	Methyl acetate	7.00	**0.22**	**3.07E-03**	**7.32E-27**	**-0.77**	≤20	≤4.3E-01
11	Ethylene carbonate	**0.10**	**12.92**	**1.25E+01**	**8.16E-09**	**-0.24**	≤20	≤3.0E+01
12	Chloroform	215.16	≤0.05	≤2.24E-05	≤1.53E-37	≤ -1.09	≤20	≤1.4E-02
13	Tetrachloro-ethene	7.00	≤0.05	≤6.89E-04	≤4.21E-30	≤ -0.87	≤20	≤4.3E-01
14	HFE 7100	238.19	≤0.05	≤2.03E-05	≤9.22E-38	≤ -1.09	≤20	≤1.3E-02
15	Dimethyl-sulfoxide	0.05	**467.02**	**9.02E+02**	**1.61E+01**	**-**	**53**	**1.6E+02**
16	n-Methyl-pyrrolidone	0.05	**40.04**	**7.73E+01**	**7.45E-05**	**-0.12**	≤20	≤6.0E+01
17	Isopropanol	1.00	≤0.05	≤4.83E-03	≤7.07E-26	≤ -0.74	≤20	≤3.0E+00
18	Chlorobenzene	7.00	≤0.05	≤6.89E-04	≤4.21E-30	≤ -0.87	≤20	≤4.3E-01
19	Dichlorobenzene	8.00	≤0.05	≤6.03E-04	≤2.16E-30	≤ -0.88	≤20	≤3.8E-01
20	Hexane	5.00	≤0.05	≤9.65E-04	≤2.29E-29	≤ -0.85	≤20	≤6.0E-01
21	Benzene	10.00	≤0.05	≤4.83E-04	≤7.07E-31	≤ -0.89	≤20	≤3.0E-01
22	Ethyl acetate	6.50	**0.64**	**9.57E-03**	**2.17E-24**	**-0.70**	≤20	≤4.6E-01
23	Toluene	7.00	≤0.05	≤6.89E-04	≤4.21E-30	≤ -0.87	≤20	≤4.3E-01
*24**	*Water*	*1.00*	***51.75***	***4.99E+00***	***8.37E-11***	***-0.30***	***49***	***7.4E+00***
*25**	*Pyridine*	*0.05*	***16.46***	***3.18E+01***	***8.74E-07***	***-0.18***	*≤20*	*≤6.0E+01*
*26**	*Diethyl carbonate*	*5.50*	*≤0.05*	*≤8.78E-04*	*≤1.40E-29*	*≤ -0.85*	*≤20*	*≤5.5E-01*

* *The precipitate in these solvents did not become orange, indicating that $CsPbBr_3$ is not the most stable crystal phase. Therefore, these solvents will not result in stable electrochemistry on $CsPbBr_3$ due to an expected phase change.*

SI-15 – ICP-OES Analysis of Bulk $CsPbBr_3$ Solubility – Graphs

Figures S-10 shows the K_{sp} and expected electrochemical stabilization of $CsPbBr_3$ in the solvents listed in Table S-1 (SI-14). In general, higher $CsPbBr_3$ solubilities are observed in more polar solvents. For most low-polarity solvents, the Pb concentration was below the detection limit of ICP-OES. Solvents generally used to dissolve perovskite-precursors, such as DMF, DMSO, and NMP (solvent numbers 9, 15 and 16 respectively) understandably exhibit the highest solubility and hence the lowest electrochemical stabilization for $CsPbBr_3$.

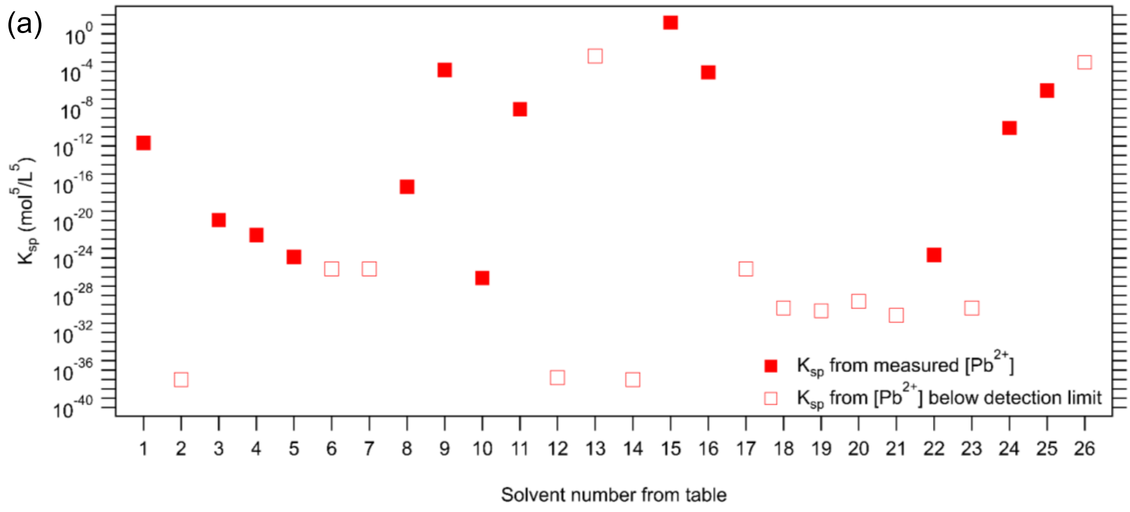

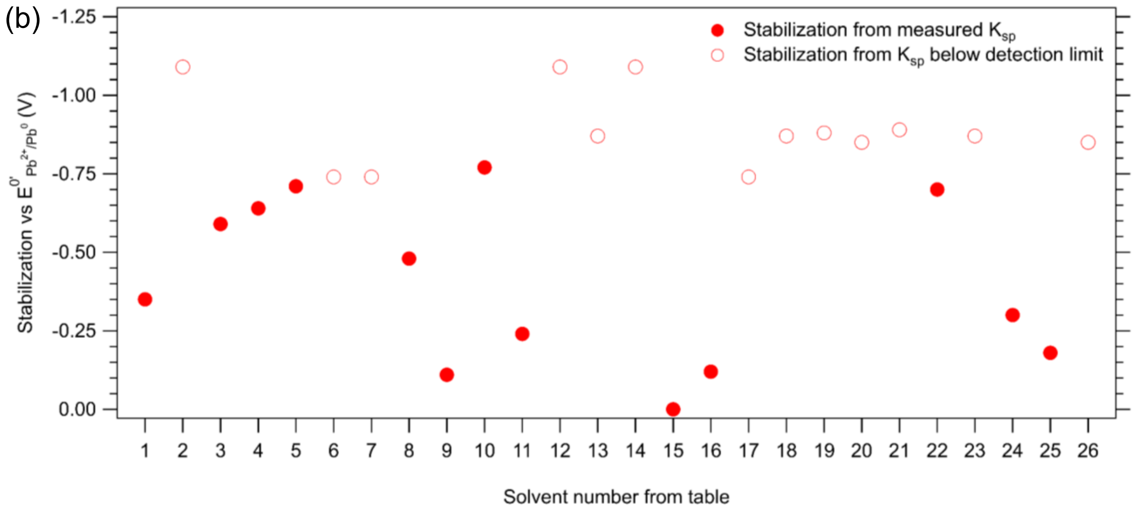

Figure S-10. (a) The determined K_{sp} values for bulk $CsPbBr_3$ in the solvents listed in Table S-1. Filled markers indicate K_{sp} values based on measured Pb concentrations above the detection limit. Samples with a Pb concentration below the detection limit are represented by empty square markers, resembling K_{sp} values that are equal to or lower than the indicated values. (b) The expected electrochemical stabilization of $E^{0'}_{CsPbBr_3/Pb}$ *versus* $E^{0'}_{Pb^{2+}/Pb}$ in solution, as determined from K_{sp} for bulk $CsPbBr_3$. Filled markers indicate the electrochemical stabilization potentials based on measured Pb concentrations above the detection limit. Samples with a Pb concentration below the detection limit are represented by empty circled markers, resembling electrochemical stabilizations that are equal to or more negative than the indicated values.

SI-16 – ICP-OES Analysis of $Pb(OA)_2$ and CsOA Solubility – Values

Table S-2. The saturation concentration of $Pb(OA)_2$ in the five solvents used throughout this work, as measured through ICP-OES of Pb. All samples were measured above the detection limit.

Solvent	Volume used (mL)	[Pb] from ICP (ppm)	Saturation [Pb] (mM)
Propylene carbonate	10.00	**10.30**	**5.00E-02**
Dichloro-methane	1.00	**684.43**	**3.30E+01**
Acetonitrile	10.00	**5.52**	**2.70E-02**
Benzonitrile	1.00	**118.04**	**5.70E+00**
Tetrahydro-furan	0.80	**1751.03**	**1.06E+02**

Table S-3. The saturation concentration of CsOA in the five solvents used throughout this work, as measured through ICP-OES of Cs. For acetonitrile and benzonitrile, where the Cs concentrations fell below the detection limit, the values corresponding to the detection limit concentration are indicated (*without* bold formatting). Therefore, the actual solubility of CsOA in these solvents is equal to or lower than the reported values.

Solvent	Volume used (mL)	[Cs] from ICP (ppm)	Saturation [Cs] (mM)
Propylene carbonate	10.00	**29**	**2.9E-01**
Dichloromethane	0.10	**65**	**6.5E-01**
Acetonitrile	19.00	≤20	≤2.5E+00
Benzonitrile	0.60	≤20	≤1.5E-01
Tetrahydrofuran	10.00	**52**	**5.2E-01**

SI-17 – OD and ΔOD Spectra of $CsPbBr_3$ Films Before and After CV Measurements

Figure S-11 shows the OD and ΔOD spectra of the $CsPbBr_3$ films before and after three CV cycles. The dashed lines indicate the wavelength regions over which is averaged to correct ΔOD for changes in the scattering intensity, as detailed in Figure S-12 (SI-18). The initial perovskite absorbance (red) of the bulk film in DCM in Figure S-11a disappeared completely after the CV measurement (black) due to cathodic decomposition. The increase in the OD baseline is explained by the formation of metallic Pb^0. A similar set of spectra was obtained for the NC film in DCM (Figure S-11b). However, for the NC film in PC (Figure S-11c), the change to the OD is more subtle. The OD spectrum after the CV measurement retained the characteristic perovskite absorbance, indicating that the $CsPbBr_3$ NCs remained largely intact. Nevertheless, the OD spectrum exhibits a slight overall decrease in intensity due to partial cathodic dissolution, along with a small increase of the baseline due to the formation of Pb^0.

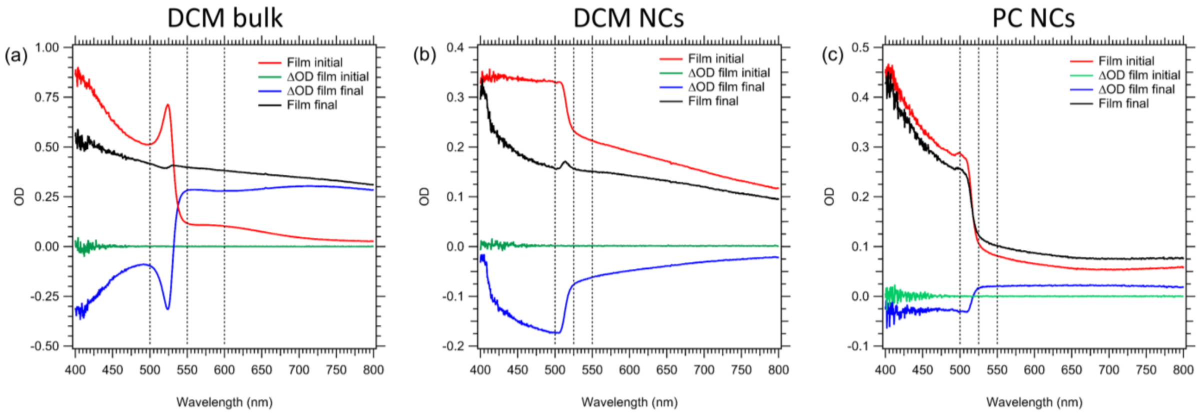

Figure S-11. (a) OD and ΔOD spectra before and after the CV measurements were performed on a bulk $CsPbBr_3$ film and (b) a NC film in DCM, and (c) a NC film in PC, with 0.1 M $TBAPF_6$ supporting electrolyte. This figure shows the near complete decomposition of both films in DCM, and only minor changes to the film in PC.

SI-18 – Correction to Perovskite OD in CV Measurements

As explained in the main text, the data presented in Figure 4d-f was obtained by a correcting the measured OD for the sub-bandgap changes arising from electrodeposition of Pb^0 on the electrode. For example, for the bulk film in DCM shown in Figure 4a in the main text, we subtract the ΔOD signal averaged between 550 nm and 600 nm from the ΔOD signal averaged between 500 nm and 550 nm (dashed vertical lines in Figure 4a of the main text). This correction provides a ΔOD signal that is specific for changes of the perovskite absorbance only. Here, we work under the assumption that the baseline offset at sub-bandgap wavelengths is similar at the band-edge of the perovskite. Figure S-12 displays the change in the perovskite absorbance (black), the change in scattering intensity at sub-bandgap wavelengths (blue), and the combined effect of these two factors (red).

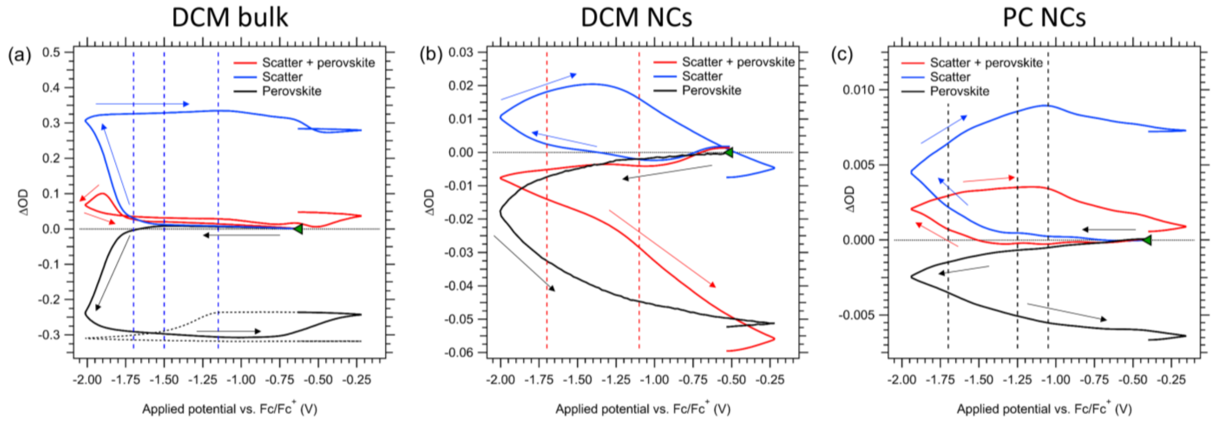

Figure S-12. (a) The ΔOD of the perovskite (as used for Figure 4d-f in the main text), obtained by subtracting the changes in the sub-bandgap scattering intensity (blue) from the band edge absorbance region (red) for the bulk film in DCM, (b) the NC film in DCM, and (c) the NC film in PC. Dashed lines denote the potentials discussed in the main text.

SI-19 – Correlation of Injected Charge to Change of OD for NC Samples

As explained in the main text, the spectro-electrochemical responses of the NC films in DCM and PC are notably different. In Figure S-13a we show that for DCM, the injected charge in the first CV cycle is roughly proportional to the change of the perovskite OD, suggesting that the injected charge is predominantly used for the cathodic decomposition reaction. In Figure S-13b, we show that in PC, the OD is more affected by gradual dissolution, as charge extraction and charge injection both result in a similar reduction in perovskite OD.

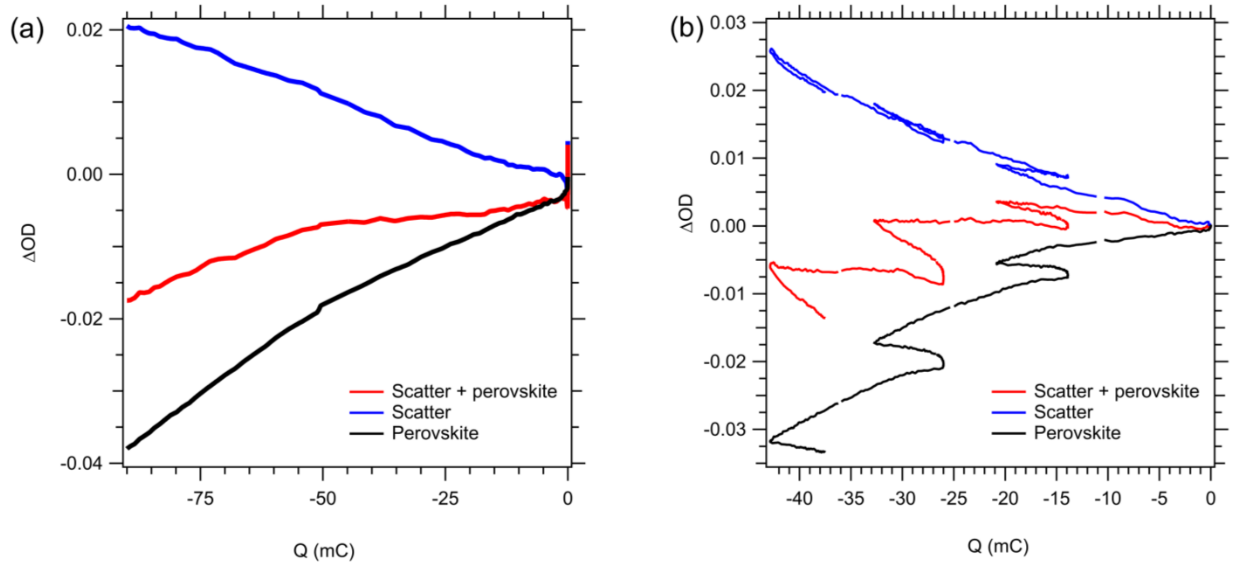

Figure S-13. (a) The ΔOD of the perovskite NCs as function of the injected charge (Q) for the first CV cycle in DCM, and (b) for all three CV cycles in PC.

SI-20 – Correlation of the PL-peak Position to the Applied Potential and Time

Figure 5c of the main text shows a blueshift of the PL-peak position of $CsPbBr_3$ NCs during the CV measurement in DCM. To obtain insight whether the blueshift is caused by electrochemical degradation or by dissolution of the film, the change of PL-peak position was plotted as function of the applied potential and time (Figure S-14). This figure shows that at potentials below the Pb^{2+}-reduction potential the blueshift occurs somewhat faster. However, even at the most positive applied potentials, a blueshift is observed, suggesting that the NC film dissolved over time. This observation is in agreement with the time-correlated PL-peak position, which shows more of a gradual decrease over time, as is expected for dissolution of the NCs.

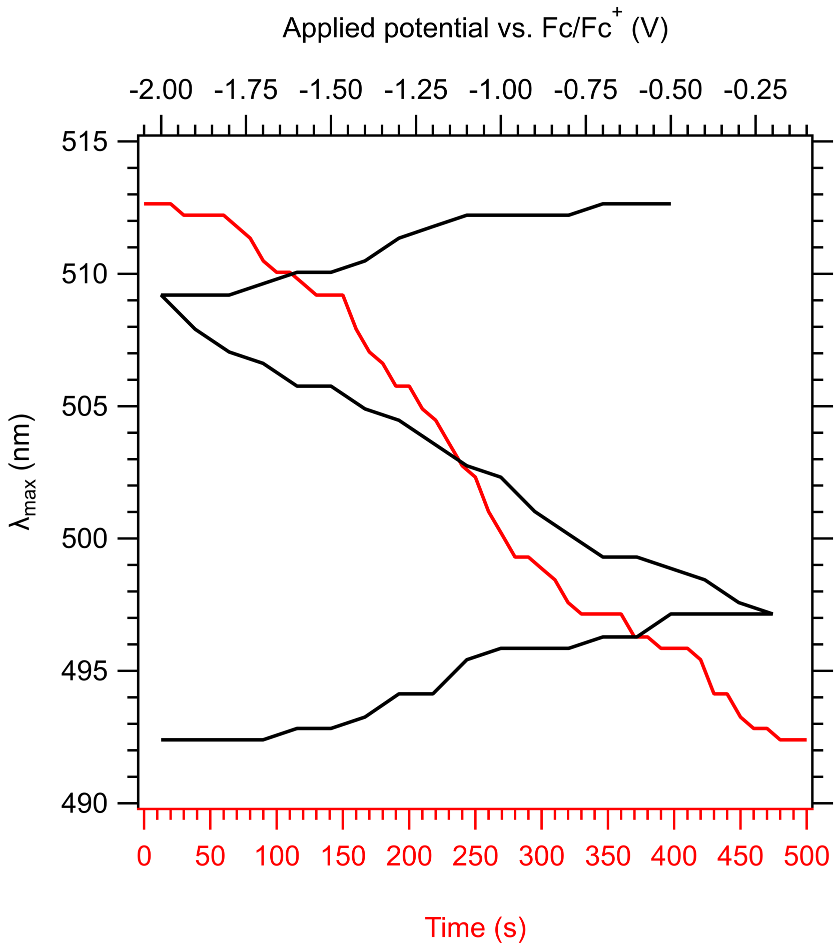

Figure S-14. The PL-peak position of the NC film in DCM with 0.1 M $TBAPF_6$ as function of the applied potential and the time. t_0 = the start of the CV measurement.

SI-21 – Electrochemistry – Lead Bromide – PC

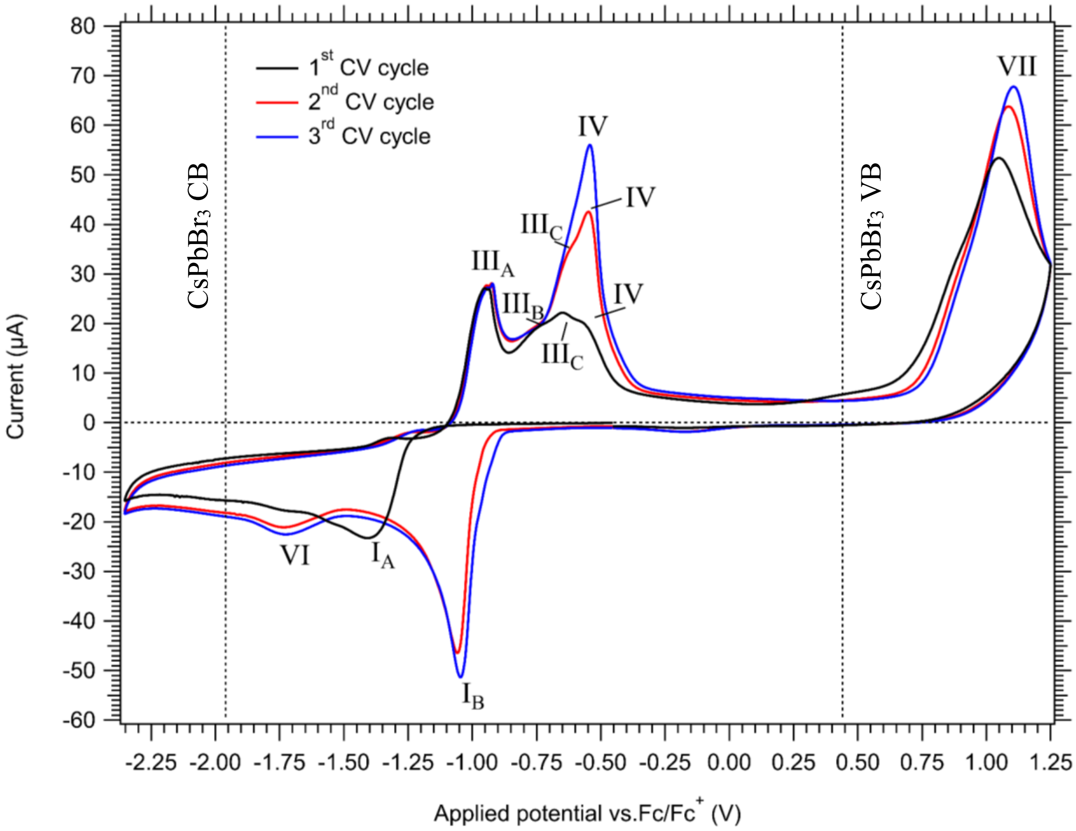

Figure S-15. A CV of a saturated solution of $PbBr_2$ in PC with 0.1 M $TBAPF_6$ on a blank ITO electrode. In accordance with the CV of a solution of $CsPbBr_3$ in PC (Figure 2c in the main text), Pb^{2+} complexes in solution are reduced in cathodic waves I and VI, and re-oxidized in anodic waves III and IV. Additionally, oxidation of Br^- is observed at anodic wave VII. As mentioned in the main text, this reaction takes place at more positive potentials than the valence band (VB) edge of $CsPbBr_3$.

I: $Pb^{2+} + 2\ e^- \rightarrow Pb^0$

III: $Pb^0 + n\ Br^- \rightarrow [PbBr_n]^{(2-n)} + 2\ e^-$ $(n_1 > n_2 > n_3)$

IV: $Pb^0 \rightarrow Pb^{2+} + 2\ e^-$

VI: $[PbBr_n]^{(2-n)} + 2\ e^- \rightarrow Pb^0 + n\ Br^-$

VII: $2\ Br^- \rightarrow Br_2 + 2\ e^-$

SI-22 – $CsPbBr_3$ Nanocrystal Electrochemistry – MeCN

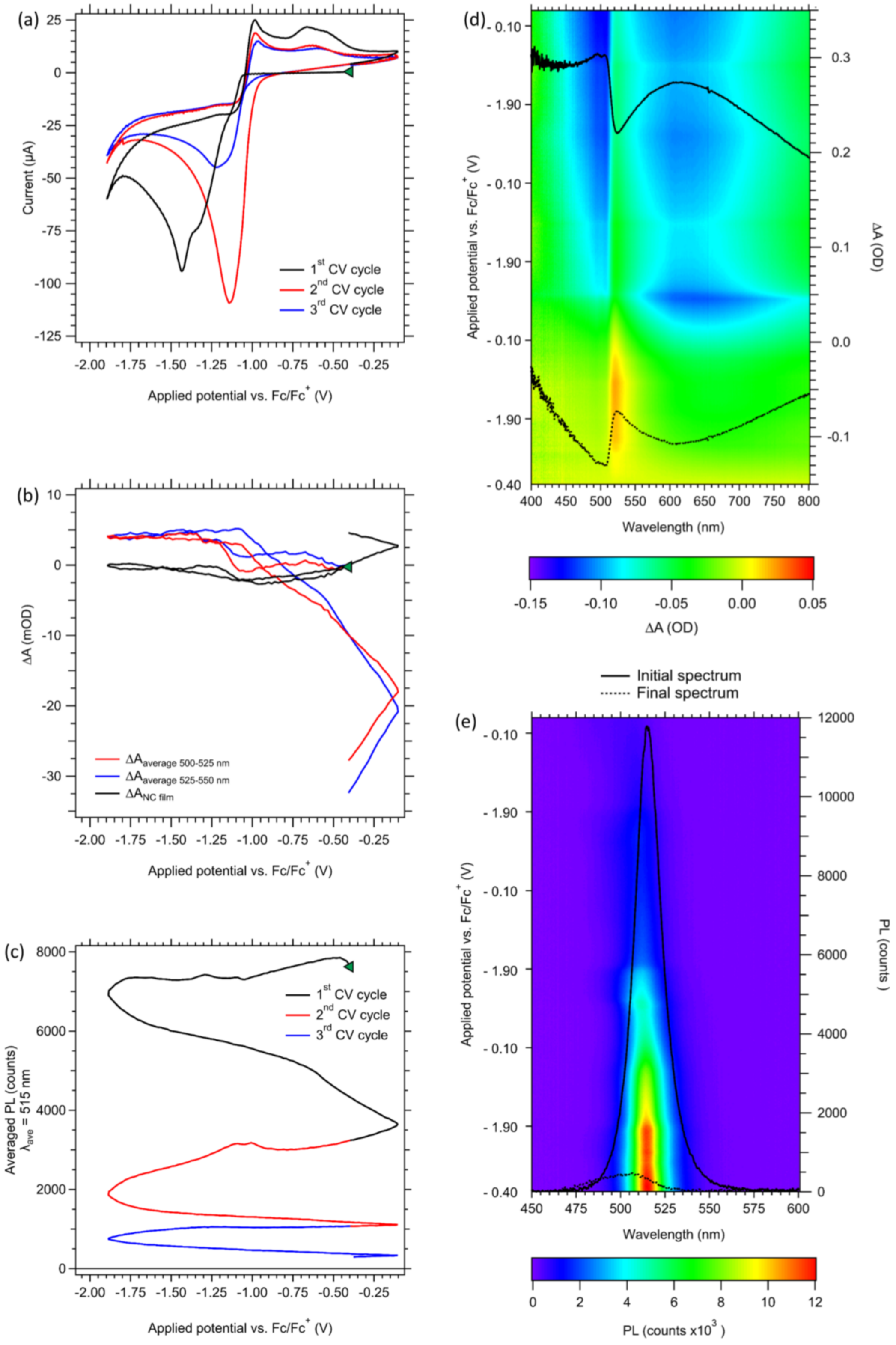

Figure S-16. Spectro-electrochemical dataset of a measurement on a $CsPbBr_3$ NC thin film in acetonitrile with 0.1 M $TBAPF_6$ as supporting electrolyte and a scan rate of 10 mV/s. (a) The CV, (b) the change of the OD in the first CV cycle, (c) the PL-intensity during the three CV cycles, and (d) 2D false-color images showing the change in the OD and (e) the PL.

SI-23 – $CsPbBr_3$ Nanocrystal Electrochemistry – PhCN

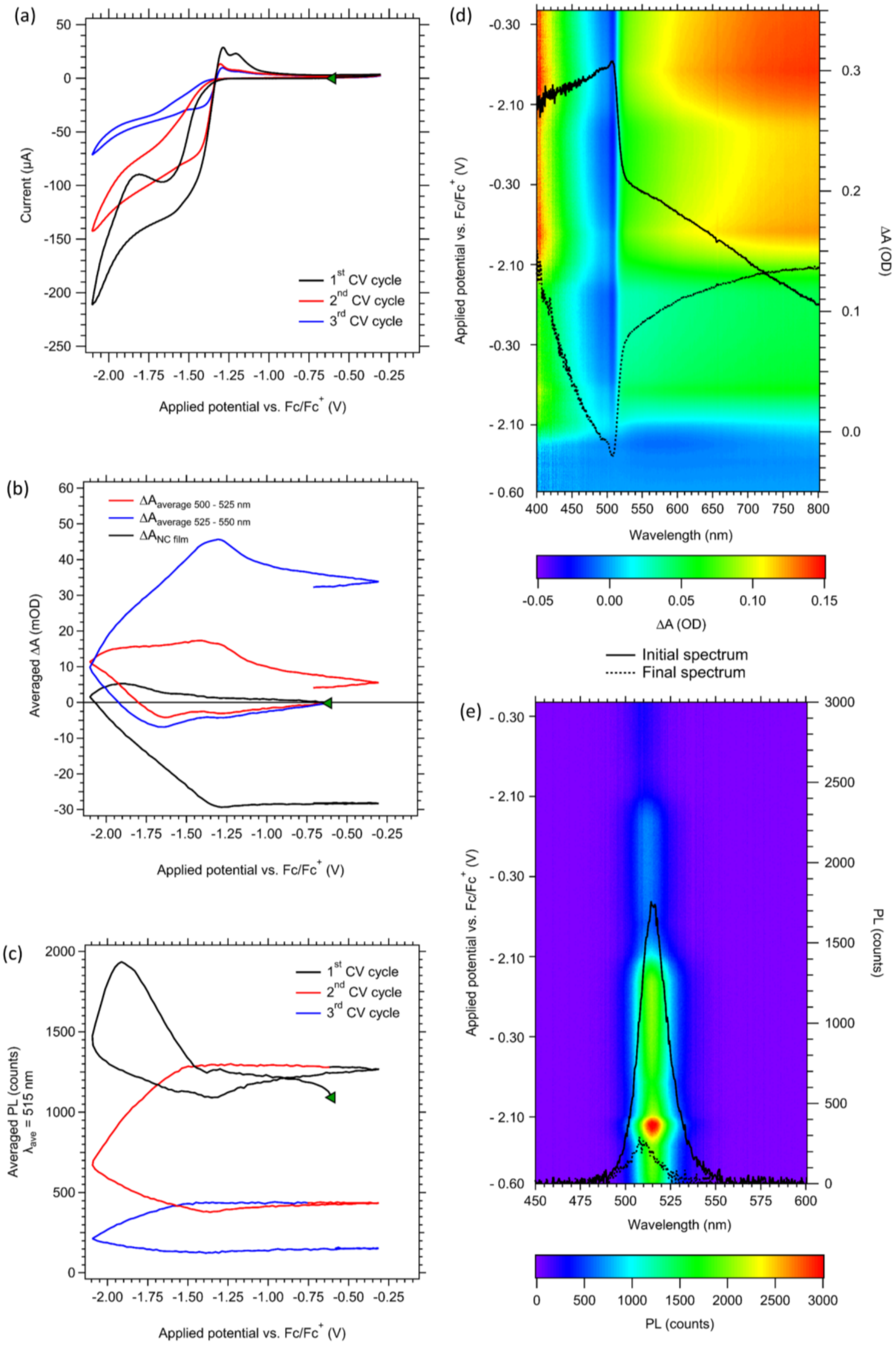

Figure S-17. Spectro-electrochemical dataset of a measurement on a $CsPbBr_3$ NC thin film in benzonitrile with 0.1 M $TBAPF_6$ as supporting electrolyte and a scan speed of 10 mV/s. (a) The CV, (b) the change of the OD in the first CV cycle, (c) the PL-intensity during the three CV cycles, and (d) 2D false-color images showing the change in the OD and (e) the PL.

SI-24 – $CsPbBr_3$ Nanocrystal Electrochemistry – THF

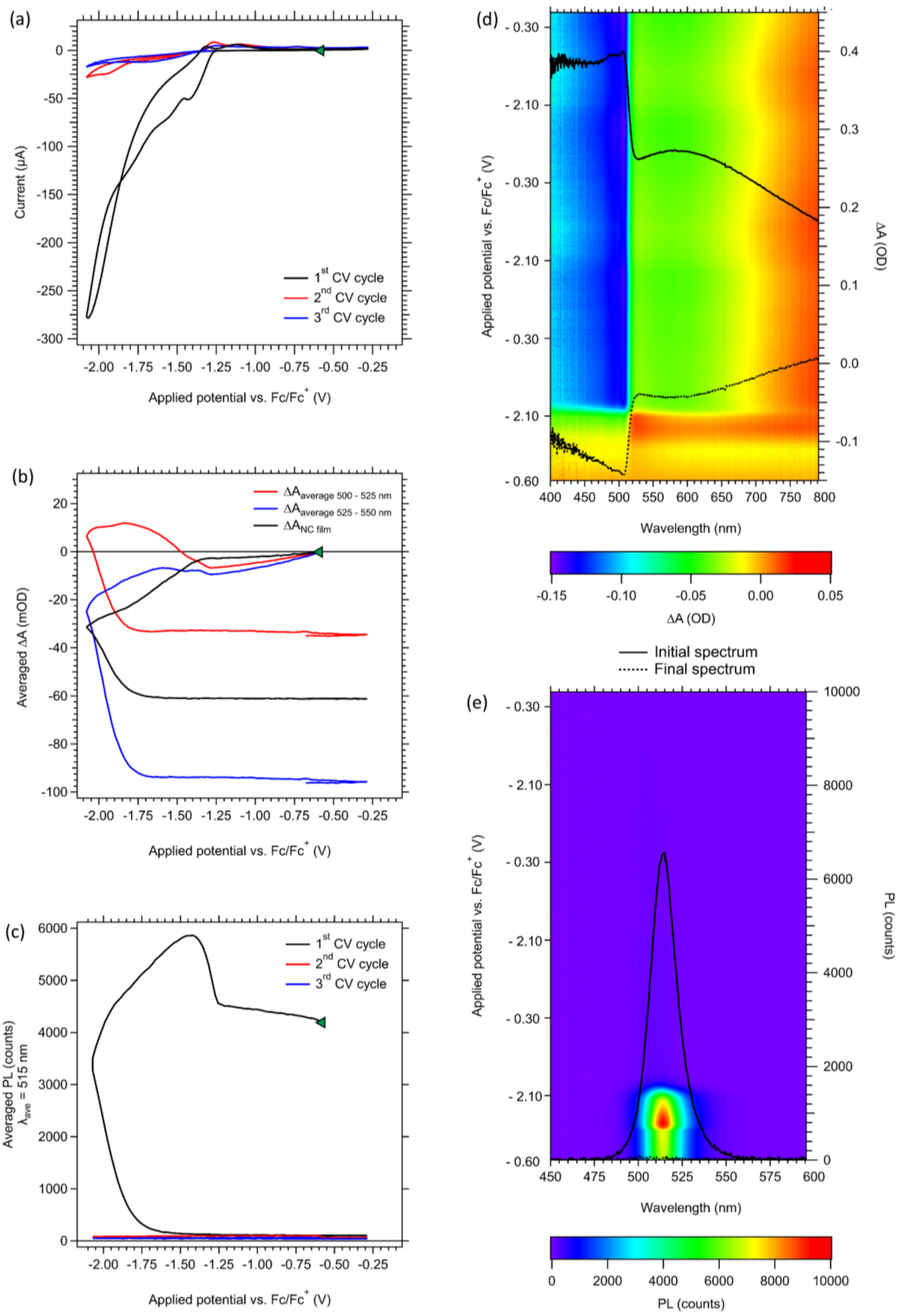

Figure S-18. Spectro-electrochemical dataset of a measurement on a $CsPbBr_3$ NC thin film in tetrahydrofuran with 0.1 M $TBAPF_6$ as supporting electrolyte and a scan speed of 10 mV/s. (a) The CV, (b) the change of the OD in the first CV cycle, (c) the PL-intensity during the three CV cycles, and (d) 2D false-color images showing the change in the OD and (e) the PL.

SI-25 – Electrochemistry – Background Current in Blank CVs of the Used Electrolytes

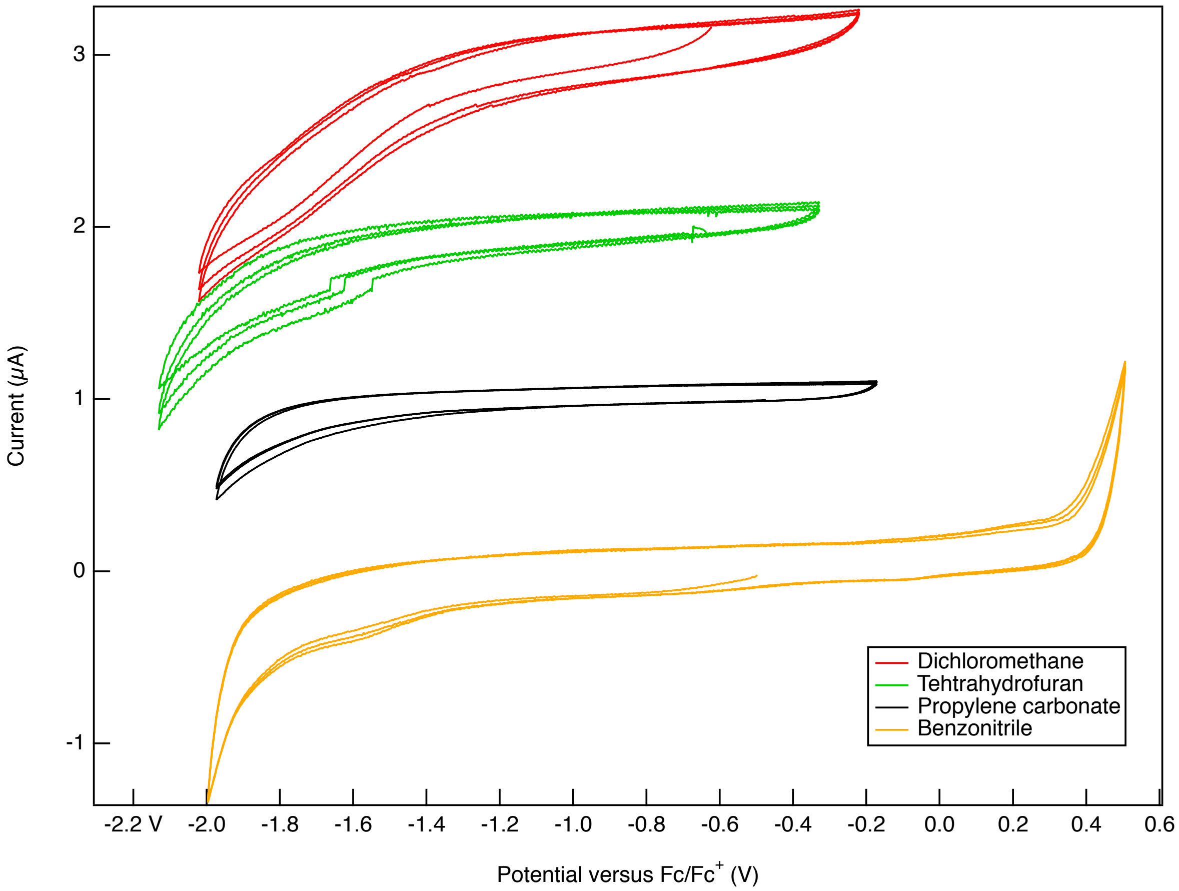

Figure S-19: Blank CVs of DCM, THF, PC and PhCN with 0.1 M $TBAPF_6$ supporting electrolyte. The individual traces are vertically offset by 1 μA for visual reference. The CVs were measured on an ITO electrode at a scan rate of 10 mV s^{-1}.

SI-26 – Stability of $CsPbBr_3$ Nanocrystal Thin Films

As mentioned in the main text, the NC thin films are not entirely stable in PC (with 0.1 M $TBAPF_6$) because of the large $CsPbBr_3$ solubility. However, due to the very insoluble ligand shell in PC, the dissolution rate of the NCs is significantly slower compared to bulk $CsPbBr_3$. As shown in Figure S-20, approximately 75% of the film remained after 1800 seconds when no potential is applied.

When TBABr is added to the electrolyte solution, the solubility of the NCs increases as the equilibrium is driven towards the formation of Pb^{2+} complexes with Br^{-} ions that are more soluble. Consequently, as depicted in Figure S-20, the NC films exhibit a faster decrease of the absorbance as the TBABr concentration in the electrolyte increases, indicating a reduced stability against dissolution of the films. Notably, for a solution containing 100 mM $TBAPF_6$ together with 1000 mM TBABr, the film completely dissolved almost instantaneously upon contact.

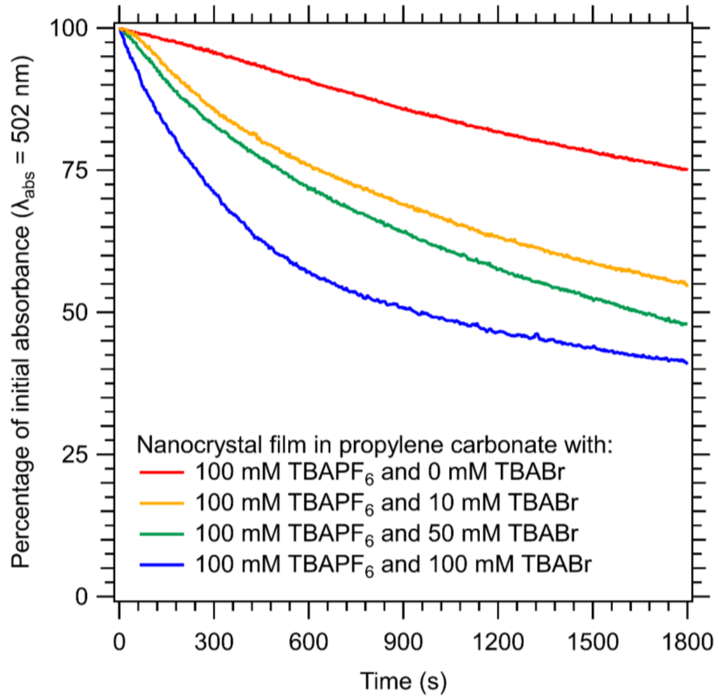

Figure S-20. The stability of the $CsPbBr_3$ NC thin films in PC with 100 mM $TBAPF_6$ and various concentrations of TBABr. All trends are made by measuring the change of the absorbance intensity at the band-edge (502 nm) relative to the initial absorbance.

SI-27 – OD Spectra of $CsPbBr_3$ Films at Different Potentials during CV Measurements

Figure S-21 shows the OD spectra of the $CsPbBr_3$ films during the CV measurements discussed in the main text. The initial perovskite absorbance (red) of the bulk film in DCM in Figure S-21a decreased significantly during the first cycle. The increase in the OD baseline is explained by the formation of metallic Pb^0. For the NC film in DCM, the decrease in the perovskite absorbance is accompanied by a blue shift during the first cathodic scan. This aligns with the observed blue-shift of the PL (shown in Figure 5c in the main text), suggesting that the NCs are etched due to an electrochemical surface reaction. As for the NC film in PC (Figure S-21c), no blue shift is observed, and the change to the OD is subtle. The OD spectrum after the CV measurement retained the characteristic perovskite absorbance, indicating that the $CsPbBr_3$ NCs remained largely intact.

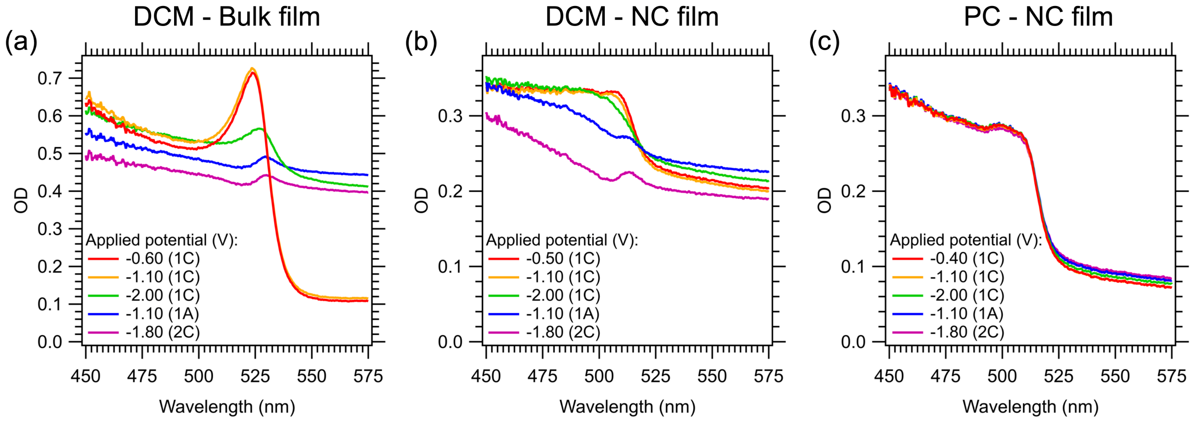

Figure S-21. OD spectra of (a) a bulk film in DCM, (b) a NC film in DCM and (c) a NC film in PC at specific potentials during the first cathodic cycle (1C), first anodic cycle (1A), and second cathodic cycle (2C) of the CV measurements. All measurements were taken with 0.1 M $TBAPF_6$ supporting electrolyte.

References

1. Imran, M.; Caligiuri, V.; Wang, M.; Goldoni, L.; Prato, M.; Krahne, R.; De Trizio, L.; Manna, L. Benzoyl Halides as Alternative Precursors for the Colloidal Synthesis of Lead-Based Halide Perovskite Nanocrystals. *J. Am. Chem. Soc.* **2018**, *140* (7), 2656–2664.
2. Hendricks, M. P.; Campos, M. P.; Cleveland, G. T.; Jen-La Plante, I.; Owen, J. S. A Tunable Library of Substituted Thiourea Precursors to Metal Sulfide Nanocrystals. *Science* **2015**, *348* (6240), 1226–1230.
3. Bohn, B. J.; Tong, Y.; Gramlich, M.; Lai, M. L.; Döblinger, M.; Wang, K.; Hoye, R. L. Z.; Müller-Buschbaum, P.; Stranks, S. D.; Urban, A. S.; Polavarapu, L.; Feldmann, J. Boosting Tunable Blue Luminescence of Halide Perovskite Nanoplatelets through Postsynthetic Surface Trap Repair. *Nano Lett.* **2018**, *18* (8), 5231–5238.
4. Mulder, J. T.; du Fossé, I.; Alimoradi Jazi, M.; Manna, L.; Houtepen, A. J. Electrochemical P-Doping of $CsPbBr_3$ Perovskite Nanocrystals. *ACS Energy Lett.* **2021**, *6* (7), 2519–2525.
